# Differential gene expression in femoral bone from red junglefowl and domestic chicken, differing for bone phenotypic traits

**DOI:** 10.1186/1471-2164-8-208

**Published:** 2007-07-02

**Authors:** Carl-Johan Rubin, Johan Lindberg, Carolyn Fitzsimmons, Peter Savolainen, Per Jensen, Joakim Lundeberg, Leif Andersson, Andreas Kindmark

**Affiliations:** 1 Department of Medical Sciences, Uppsala University, Sweden; 2 Department of Gene Technology, School of Biotechnology, Royal Institute of Technology, Stockholm, Sweden; 3 Department of Animal Breeding and Genetics, Swedish University of Agricultural Sciences, Uppsala, Sweden; 4 IFM Biology, Linköping University, SE-585 83, Linköping, Sweden; 5 Department of Medical Biochemistry and Microbiology, Uppsala University, Box 597, SE-75124 Uppsala, Sweden

## Abstract

**Background:**

Osteoporosis is frequently observed among aging hens from egg-producing strains (layers) of domestic chicken. White Leghorn (WL) has been intensively selected for egg production and it manifests striking phenotypic differences for a number of traits including several bone phenotypes in comparison with the wild ancestor of chicken, the red junglefowl (RJ). Previously, we have identified four Quantitative Trait Loci (QTL) affecting bone mineral density and bone strength in an intercross between RJ and WL. With the aim of further elucidating the genetic basis of bone traits in chicken, we have now utilized cDNA-microarray technology in order to compare global RNA-expression in femoral bone from adult RJ and WL (five of each sex and population).

**Results:**

When contrasting microarray data for all WL-individuals to that of all RJ-individuals we observed differential expression (False discovery rate adjusted p-values < 0.015) for 604 microarray probes. In corresponding male and female contrasts, differential expression was observed for 410 and 270 probes, respectively. Altogether, the three contrasts between WL and RJ revealed differential expression of 779 unique transcripts, 57 of which are located to previously identified QTL-regions for bone traits. Some differentially expressed genes have previously been attributed roles in bone metabolism and these were: WNT inhibitory factor 1 (WIF1), WD repeat-containing protein 5 (WDR5) and Syndecan 3 (SDC3). Among differentially expressed transcripts, those encoding structural ribosomal proteins were highly enriched and all 15 had lower expression in WL.

**Conclusion:**

We report the identification of 779 differentially expressed transcripts, several residing within QTL-regions for bone traits. Among differentially expressed transcripts, those encoding structural ribosomal proteins were highly enriched and all had lower expression levels in WL. In addition, transcripts encoding four translation initiation and translation elongation factor proteins also had lower expression levels in WL, possibly indicating perturbation of protein biosynthesis pathways between the two populations. Information derived from this study could be relevant to the bone research field and may also aid in further inference of genetic changes accompanying animal domestication.

## Background

Osteoporosis is a complex multifactorial disease that constitutes a major public health problem in our steadily aging human population. The disease is characterized by an altered bone metabolism leading to reduced bone mineral density (BMD) and degeneration of bone tissue, and thus an increase in bone fragility and risk of fracture. It has been estimated that every other female, but also every fourth male will sustain an osteoporotic fracture during their lifetime [1]

Bone metabolism is complex, influenced by genetic, environmental and life style factors. Twin studies show that genetic factors play an important role in the development of different skeletal phenotypes coupled to fractures and even to the incidence of fracture itself. For example, genetic factors have been estimated to account for 70–80% of the variance in bone mineral density (BMD) [2,3] and for 25–35% of the risk for fracture [4]. A multitude of genes, some of which remain to be identified are involved in bone metabolism, and normal inter-individual variation in traits such as BMD and bone strength are thus most likely dependent on the combined subtle effects of many alleles.

Chicken *(Gallus gallus) *has traditionally been used as a model species in various branches of biology such as embryology, immunology, behavior and reproductive research [5]. The recently released draft sequence of the chicken genome [6] has made chicken an attractive model species also for genetic studies. The size of the chicken genome is about one third of the human genome size but with approximately the same number of genes and therefore has a higher gene density, which facilitates genetic studies. Furthermore, birds possess a unique evolutionary position within the vertebrate lineage, and are therefore particularly interesting for comparative genomics.

The red junglefowl (RJ) is the wild ancestor of domestic chicken. Domestication of chicken is believed to have been initiated in South-East Asia several thousand years ago [7], although several additional events of domestication may have occurred more recently. Selection in domestic breeds for traits beneficial in meat and egg production has manifested great phenotypic differences in body composition between wild-type and domestic chicken, and between domestic breeds. The phenotypic heterogeneity present among extant chicken populations presents opportunities for the identification of genetic elements on which selection has acted.

In chicken as well as in man, cortical and trabecular bone provide the mineralized framework enabling locomotion through bones acting as levers for muscles. Bone tissue provides structural support and protection for internal organs and has various metabolic functions. In the sexually mature female bird, dietary calcium and skeletal hydroxyapatite are both utilized as sources of calcium for deposition in eggshells. Elevation in levels of estrogen shortly before the onset of egg laying stimulates formation of a bone type called medullary bone, which throughout the egg-laying period acts as a labile calcium store mobilized to form eggshells [8]. Medullary and cortical bone are both resorbed in response to egg-laying, but since medullary bone is remodeled at a much higher rate than cortical bone there is a decline in cortical bone mass during the productive period [9]. Subsequent cortical bone loss results in bone fragility due to low amounts of structural bone and at the end of lay and osteoporosis is frequently observed in hens from commercial egg-production facilities [10]. In chicken as well as in man, osteoporosis is a disease manifested by bone fragility late in life, but the functional aspects of the disease differ between the two species. In chicken the major mechanism appears to be significant remodeling of medullary bone occurring at the expense of cortical bone remodeling during long productive periods when levels of estrogen are high. This is in contrast to the human situation, where the post-menopausal decline in estrogen is acknowledged as an important contributing factor to the development of disease. Thus, the process by which the disease develops is likely to differ between man and chicken. In man, low peak bone mass is a strong positive predictor for the development of osteoporosis and therefore it is important to identify genetic variants governing peak bone mass. The main objective of this study was to identify genes which are responsible for differences in peak bone mass in chicken; as such genes could prove important to bone metabolism also in man.

White Leghorn (WL) is a domestic breed which during the last century has been intensely selected for egg production and consequently WL and RJ exhibit large differences in bone phenotypes, with BMD differing as much as 50% between hens of the two populations at peak bone mass [11]. In a three-generation intercross between WL and wild-type red junglefowl (RJ), we have previously identified four significant and several suggestive Quantitative Trait Loci (QTL) affecting various traits of femoral bone [11].

In man and in animal models, many loci with effects on bone traits have been identified by linkage- and QTL-analyses (reviewed in [12]). However, the path from QTL to the identification of causative genes has often proven difficult. A commonly used approach in animal models is repeated backcrossing to generate congenic lines, but even with such efforts, QTL-intervals are often broad, containing multiple candidate genes. Genetic differences causative of QTLs may affect gene expression, either through cis-effects on transcription of adjacent genes [13,14] or through trans-acting effects, whereby transcription from loci not necessarily in the chromosomal vicinity of the QTL may be affected. Therefore, global gene expression profiling in parental populations may provide crucial information on QTL effects that facilitates the identification of the causal gene.

In this study, gene expression profiling and QTL-analysis have been combined, as these together could facilitate identification of molecular pathways and ultimately genes whose perturbation has resulted in bone phenotypic variation. As the chickens studied are at the age of peak bone mass, this study is primarily aimed at identifying loci contributing to bone acquisition rather than to bone loss and osteoporosis. Information derived from the present study may provide novel insights regarding the genetic regulation of bone tissue in vertebrates and could enable discovery of alleles conferring high or low BMD and thus novel targets for pharmacological prevention of osteoporosis.

## Results

### B-test for differential expression

**Figure 1:**
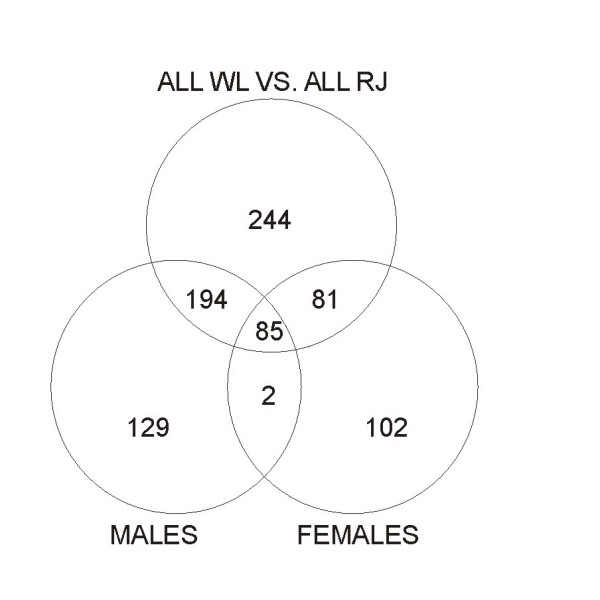
Venn diagram indicating numbers of probes sharing differential expression (q-values < 0.015) in sex specific contrasts between WL and RJ and in contrast between all WL and all RJ). Numbers within shared fields of circles indicate the number of probes exhibiting DE in overlapping contrasts.

**Figure 2:**
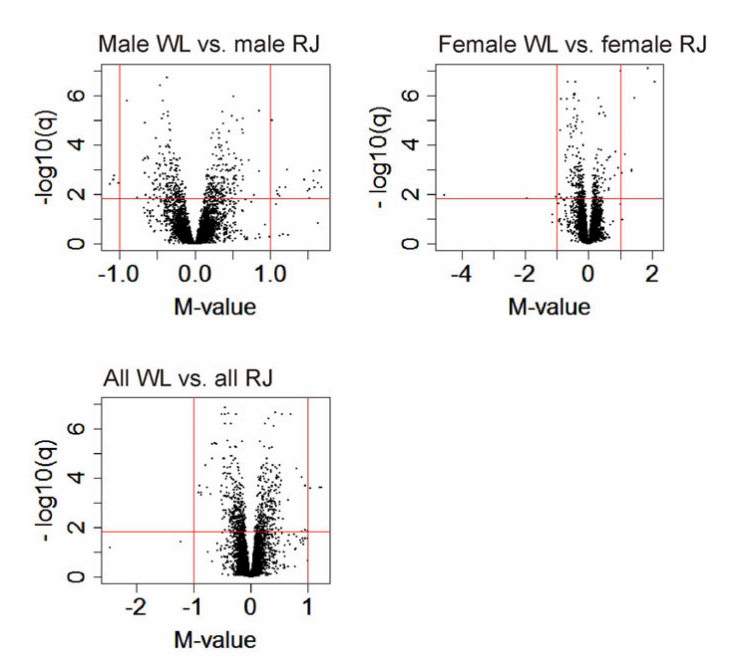
Volcano plots illustrating distributions of M- and -log(FDR-adjusted p-values) for all probes on microarray in three contrasts between WL and RJ. Horizontal red lines indicate DE significance threshold (q = 0.015) and vertical red lines indicate M-values of 1 and -1. Positive M-values indicate higher expression in WL.

**Figure 3:**
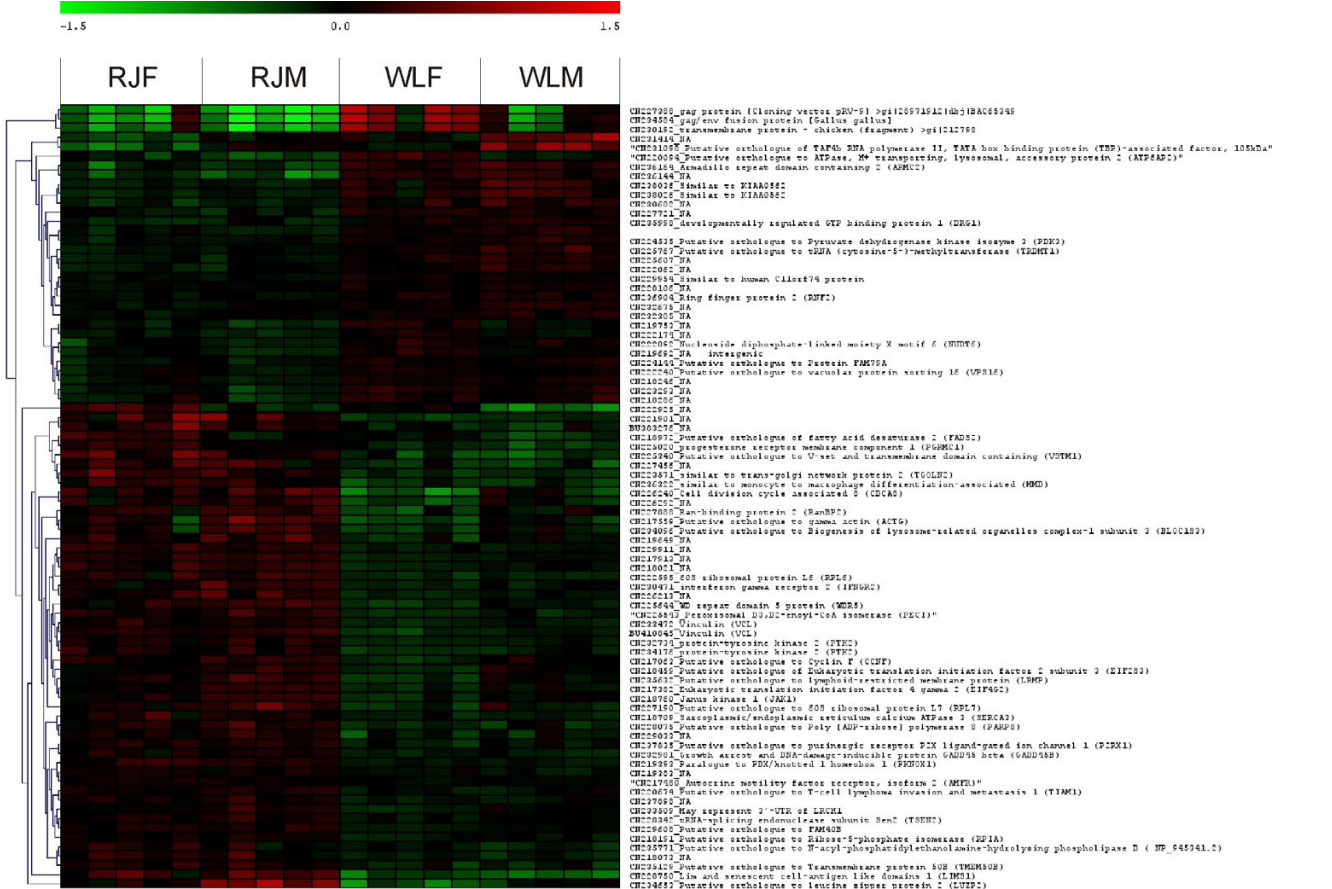
Hierarchical clustering of microarray data for 85 microarray probes showing differential expression in all three contrasts performed between red junglefowl and White Leghorn. Individuals (five of each sex and line) are scattered along the x-axis and probes are distributed along the y-axis. For each individual and probe, colors represent log2(sample fluorescence/reference fluorescence) according so upper scale bar (red indicates more expression in sample channel whereas green indicate more expression in reference channel). Hierarchical clustering was performed using the Euclidean metric.

The significance threshold for differential expression (DE) was set at False Discovery Rate (FDR) adjusted p-values (q-values) = 0.015. In Table 1 numbers and details of DE probes identified in eight separate contrasts are summarized. When all RJ-individuals were compared to all WL-individuals, DE was observed for 604 probes. DE between males of the two strains was observed for 410 probes and the corresponding female comparison revealed DE for 270 probes. A total number of 837 probes, corresponding to 779 unique transcripts were identified as DE in any of the three latter contrasts (Additional File 1) and overlap of DE between contrasts is presented as a Venn-diagram in Figure 1. Volcano plots in Figure 2 illustrate global distributions of log2(fold change in expression) (M-values) and q-values for all three contrasts performed between RJ and WL. Details regarding the 85 probes for which DE was observed in all three contrasts are presented in Table 2 and hierarchical clustering of these is presented as a heat map in Figure 3. Hierarchical clustering was also performed for all 837 probes showing DE in any contrast between the two chicken strains (Additional File 2). Close to two thirds (63%) of the probes showing DE-between females of the two strains had lower expression in WL females, which is a highly significant bias (χ_2_, d.f. = 1, P < 0.001). There was no apparent difference in the direction of differential expression between males where 48% of probes showed lower expression in WL.

**Table 1:** Numbers of probes for which differential expression (q-values < 0.015) was observed in all contrasts performed.

**Comparison**	**Number of DE-transcripts **	**More expression in RJ (%)**	**More expression in males (%)**
WL vs. RJ	604	56	NA
WLM vs. RJM	410	48	NA
WLF vs. RJF	270	63	NA
WLM vs. WLF	2769	NA	61
RJM vs. RJF	1492	NA	56
WLM vs. RJF	2164	45	55
WLF vs. RJM	3214	60	60
M vs. F	3372	NA	59

WLM = White Leghorn males, WLF = White Leghorn females, RJM = red junglefowl males, RJF = red junglefowl females. NA: Not applicable

**Table 2:** Top table of 85 microarray probes identified as differentially expressed (q < 0.015) in all three contrasts between White Leghorn and red junglefowl (male vs. male, female vs. female and all WL vs. all RJ)

GenBank ID	Ensembl gene ID	Position	Annotation ^1^	M ^2^	q-value ^3^
[GenBank:CN217913]	ENSGALG00000018770	Un: 28.3 Mb	NA	-0.40	3.2E-12
[GenBank:CN229911]	ENSGALG00000013482	Un: 21.4 Mb	NA	-0.38	9.8E-11
[GenBank:CN228750]	ENSGALG00000016807	1: 140.6 Mb	Lim and senescent cell-antigen like domains 1 (LIMS1)	-0.86	9.8E-11
[GenBank:CN225543]	ENSGALG00000012809	2: 67.1 Mb	Peroxisomal D3,D2-enoyl-CoA isomerase (PECI)	-0.43	2.7 E-09
[GenBank:CN223571]	ENSGALG00000014429	Un: 60.2 Mb	similar to trans-golgi network protein 2 (TGOLN2)	-0.56	5.0 E-09
[GenBank:CN219393]	ENSGALG00000008774	3: 104.9 Mb	Paralogue to PBX/knotted 1 homeobox 1 (PKNOX1)	-0.35	5.5 E-09
[GenBank:BU410845]	ENSGALG00000005079	6: 16.5 Mb	Vinculin (VCL)	-0.46	8.9 E-09
[GenBank:CN232734]	ENSGALG00000016171	2: 151.3 Mb	protein-tyrosine kinase 2 (PTK2)	-0.34	1.7 E-08
[GenBank:CN218021]	ENSGALG00000018770	Un: 28.3 Mb	NA	-0.41	2.1 E-08
[GenBank:CN225644]	ENSGALG00000002659	17: 7.9 Mb	WD repeat domain 5 protein (WDR5)	-0.45	1.4 E-07
[GenBank:CN238026]	ENSGALG00000000978	21: 889 kb	Similar to KIAA0562	0.44	2.2 E-07
[GenBank:CN234176]	ENSGALG00000016171	2: 151.4 Mb	protein-tyrosine kinase 2 (PTK2)	-0.39	2.4 E-07
[GenBank:CN236144]	ENSGALG00000003797	7: 4.5 Mb	NA	0.70	2.6 E-07
[GenBank:CN236164]	ENSGALG00000015289	3: 69.8 Mb	Armadillo repeat domain containing 2 (ARMC2)	0.56	2.6 E-07
[GenBank:CN229608]	ENSGALG00000007968	1: 641 kb	Putative orthologue to FAM40B	-0.25	2.6 E-07
[GenBank:CN236322]	ENSGALG00000003078	18: 6.0 Mb	similar to monocyte to macrophage differentiation-associated (MMD)	-0.51	2.6 E-07
[GenBank:CN225020]	ENSGALG00000020260	4: 16.7 Mb	progesterone receptor membrane component 1 (PGRMC1)	-0.44	2.7 E-07
[GenBank:CN235998]	ENSGALG00000006921	15: 9.3 Mb	developmentally regulated GTP binding protein 1 (DRG1)	0.32	3.8 E-07
[GenBank:CN224535]	ENSGALG00000016323	1: 121.5 Mb	Putative orthologue to Pyruvate dehydrogenase kinase isozyme 3 (PDK3)	0.26	6.1 E-07
[GenBank:CN233472]	ENSGALG00000005079	6: 16.5 Mb	Vinculin (VCL)	-0.34	6.1 E-07
[GenBank:CN222595]	ENSGALG00000004818	15: 6.5 Mb	60S ribosomal protein L6 (RPL6)	-0.45	6.1 E-07
[GenBank:CN238036]	ENSGALG00000000978	21: 889 kb	Similar to KIAA0562	0.40	7.9 E-07
[GenBank:CN226213]		1: 130.1 Mb	NA	-0.40	2.9 E-06
[GenBank:CN229954]	ENSGALG00000007941	5: 21.2 Mb	Similar to human C11orf74 protein	0.17	3.4 E-06
[GenBank:CN217480]	ENSGALG00000003107	11: 2.1 Mb	Autocrine motility factor receptor, isoform 2 (AMFR)	-0.15	3.4 E-06
[GenBank:CN221901]		NA	NA	-0.63	4.0 E-06
[GenBank:CN225340]	ENSGALG00000022549	Un: 49.4 Mb	Putative orthologue to V-set and transmembrane domain containing (VSTM1)	-0.67	4.3 E-06
[GenBank:CN227456]		2: 3.8 Mb	NA	-0.59	4.3 E-06
[GenBank:CN218246]	ENSGALG00000024009	5: 680 kb	NA	0.24	5.5 E-06
[GenBank:CN232981]	ENSGALG00000000212	Z: 43.0 Mb	Growth arrest and DNA-damage-inducible protein GADD45 beta (GADD45B)	-0.28	5.5 E-06
[GenBank:CN237898]		26: 4.8 Mb	NA	-0.25	5.5 E-06
[GenBank:CN230682]		Un: 33.1 Mb	NA	0.37	6.0 E-06
[GenBank:CN230471]	ENSGALG00000015972	1: 108.8 Mb	interferon gamma receptor 2 (IFNGR2)	-0.36	6.0 E-06
[GenBank:CN223293]		NA	NA	0.34	6.2 E-06
[GenBank:BU303036]	ENSGALG00000013094	2: 83.0 Mb	Dopa decarboxylase (DDC)	-0.34	1.3 E-05
[GenBank:CN218709]	ENSGALG00000001564	19: 3.3 Mb	Sarcoplasmic/endoplasmic reticulum calcium ATPase 3 (SERCA3)	-0.33	1.3 E-05
[GenBank:CN226292]		4: 51.2 Mb	NA	-0.46	1.6 E-05
[GenBank:CN226240]	ENSGALG00000022145	Un: 45.0 Mb	Cell division cycle associated 8 (CDCA8)	-0.67	1.6 E-05
[GenBank:CN237835]	ENSGALG00000001573	19: 3.3 Mb	Putative orthologue to purinergic receptor P2X ligand-gated ion channel 1 (P2RX1)	-0.30	2.0 E-05
[GenBank:CN218191]	ENSGALG00000015960	4: 89.4 Mb	Putative orthologue to Ribose-5-phosphate isomerase (RPIA)	-0.22	2.5 E-05
[GenBank:CN218073]	ENSGALG00000008285	1: 13.9 Mb	NA	-0.33	2.5 E-05
[GenBank:CN225607]		3: 134.8 Mb	NA	0.29	2.7 E-05
[GenBank:CN224144]	ENSGALG00000001024	21: 960 kb	Putative orthologue to Protein FAM79A	0.22	3.1 E-05
[GenBank:CN234653]	ENSGALG00000004414	5: 1.15 Mb	Putative orthologue to leucine zipper protein 2 (LUZP2)	-0.79	3.1 E-05
[GenBank:CN232675]	ENSGALG00000002384	7: 289 kb	NA	0.19	3.6 E-05
[GenBank:CN229033]		1: 6.3 Mb	NA	-0.35	3.6 E-05
[GenBank:CN220674]	ENSGALG00000015842	1: 107.9 Mb	Putative orthologue to T-cell lymphoma invasion and metastasis 1 (TIAM1)	-0.22	3.6 E-05
[GenBank:CN225767]	ENSGALG00000008686	2: 19.8 Mb	Putative orthologue to tRNA (cytosine-5-)-methyltransferase (TRDMT1)	0.26	3.8 E-05
[GenBank:CN228075]	ENSGALG00000014880	Z: 14.2 Mb	Putative orthologue to Poly ADP-ribose polymerase 8 (PARP8)	-0.40	3.9 E-05
[GenBank:CN236904]	ENSGALG00000004823	8: 8.4 Mb	Ring finger protein 2 (RNF2)	0.19	4.4 E-05
[GenBank:CN227721]	ENSGALG00000024253	Un: 21.8 Mb	NA	0.29	4.6 E-05
[GenBank:CN219383]		Un: 57.4 Mb	NA	-0.21	4.8 E-05
[GenBank:CN218286]		1: 16.9 Mb	NA	0.35	5.9 E-05
[GenBank:BU383276]		7: 5.02 Mb	NA	-0.45	6.2 E-05
[GenBank:CN228342]	ENSGALG00000004981	12: 5.12 Mb	tRNA-splicing endonuclease subunit Sen2 (TSEN2)	-0.20	6.5 E-05
[GenBank:CN219649]		18: 9.2 Mb	NA	-0.39	6.5 E-05
[GenBank:CN235129]	ENSGALG00000015976	1: 108.8 Mb	Putative orthologue to Transmembrane protein 50B (TMEM50B)	-0.36	6.9 E-05
[GenBank:CN222892]	ENSGALG00000011834	4: 55.1 Mb	Nucleoside diphosphate-linked moiety X motif 6 (NUDT6)	0.27	6.9 E-05
[GenBank:CN227190]	ENSGALG00000015637	2: 122.5 Mb	Putative orthologue to 60S ribosomal protein L7 (RPL7)	-0.43	8.8 E-05
[GenBank:CN235632]	ENSGALG00000014011	1: 69.3 Mb	Putative orthologue to lymphoid-restricted membrane protein (LRMP)	-0.35	9.3 E-05
[GenBank:CN232385]	ENSGALG00000009822	3: 23.0 Mb	NA	0.18	9.9 E-05
[GenBank:CN217382]	ENSGALG00000005587	5: 9.7 Mb	Eukaryotic translation initiation factor 4 gamma 2 (EIF4G2)	-0.29	1.2 E-04
[GenBank:CN222062]		11: 8.3 Mb	NA	0.26	1.4 E-04
[GenBank:CN218760]	ENSGALG00000011031	8: 29.0 Mb	Janus kinase 1 (JAK1)	-0.32	1.8 E-04
[GenBank:CN230192]		*	gi| 212798| ±	0.97	2.1 E-04
[GenBank:CN227388]		*	gi| 28971912| ±	1.24	2.5 E-04
[GenBank:CN234584]		*	gi| 118084693| ±	1.22	2.5 E-04
[GenBank:CN222174]	ENSGALG00000007378	1: 79.9 Mb	NA	0.20	2.9 E-04
[GenBank:CN219692]		23: 1.4 Mb	NA	0.24	3.0 E-04
[GenBank:CN217559]	ENSGALG00000009621	Un: 20.4 Mb	Putative orthologue to gamma actin (ACTG)	-0.53	3.5 E-04
[GenBank:CN222240]	ENSGALG00000014494	Un: 42.5 Mb	Putative orthologue to vacuolar protein sorting 16 (VPS16)	0.17	3.8 E-04
[GenBank:CN235771]	ENSGALG00000008285	1: 13.9 Mb	Putative orthologue to N-acyl-phosphatidylethanolamine-hydrolyzingphospholipase D (NP_945341.2)	-0.28	3.8 E-04
[GenBank:CN227888]	ENSGALG00000006545	Un: 33.2 Mb	Ran-binding protein 2 (RanBP2)	-0.42	3.8 E-04
[GenBank:DQ211077]	ENSGALG00000024372 *	16: 40 kb *	MHC class I antigen (Fragment)	-0.70	4.4 E-04
[GenBank:CN228106]	ENSGALG00000022764		NA	0.13	4.6 E-04
[GenBank:CN217063]	ENSGALG00000009201	14: 15.0 Mb	Putative orthologue to Cyclin F (CCNF)	-0.29	5.7 E-04
[GenBank:CN234096]	ENSGALG00000014339	Un: 11.3 Mb	Putative orthologue to Biogenesis of lysosome-related organelles complex-1 subunit 3 (BLOC1S3)	-0.42	6.0 E-04
[GenBank:CN220094]	ENSGALG00000016241	1: 115.9 Mb	Putative orthologue to ATPase, H+ transporting, lysosomal, accessory protein 2 (ATP6AP2)	0.37	9.4 E-04
[GenBank:CN219753]		20: 5.5 Mb	NA	0.25	9.9 E-04
[GenBank:CN231414]	ENSGALG00000016813	1: 141.1 Mb	NA	0.51	0.0011
[GenBank:CN233509]		1: 172.6 Mb	NA	-0.22	0.0019
[GenBank:CN218972]	ENSGALG00000007178	5: 18.0 Mb	Putative orthologue of fatty acid desaturase 2 (FADS2)	-0.32	0.0034
[GenBank:CN231098]	ENSGALG00000015122	2: 107.4 Mb	Putative orthologue of TAF4b RNA polymerase II, TATAbox binding protein (TBP)-associated factor, 105 kDa (TAF4B)	0.56	0.0046
[GenBank:CN222925]	ENSGALG00000011164	2: 33.7 Mb	NA	-0.46	0.012
[GenBank:CN218459]	ENSGALG00000016340	1: 121.7 Mb	Putative orthologue of Eukaryotic translation initiationfactor 2 subunit 3 (EIF2S3)	-0.20	0.014

1 Annotation: Gene name if available. Putative orthologues to ensemble chicken transcripts are presented when no gene name is available. NA = No annotation and is presented in cases where neither is applicable.

2 M-value: Log2(Fold difference in expression) in contrast all WL versus all RJ. Positive M-values indicate more expression in White Leghorn.

3 q-value: FDR-adjusted p-value from sex independent contrast between WL and RJ. * BLAST search of probe sequence against the chicken genome produced multiple hits± Represents endogenous retroviral elements. Highest scoring BLAST hit is presented.

### Validation of differential expression by quantitative PCR

Glyceraldehyde 3-phosphate dehydrogenase (*GAPDH*) was used as endogenous control probe for the microarray, and had been spotted in different concentrations in many separate spots. Upon examination of microarray fluorescence data one WL male was found to express *GAPDH *in levels around 50% lower than the other 19 individuals (Additional File 3). Since *GAPDH *had been chosen as reference gene, this individual was excluded from qPCR analyses.

**Figure 4:**
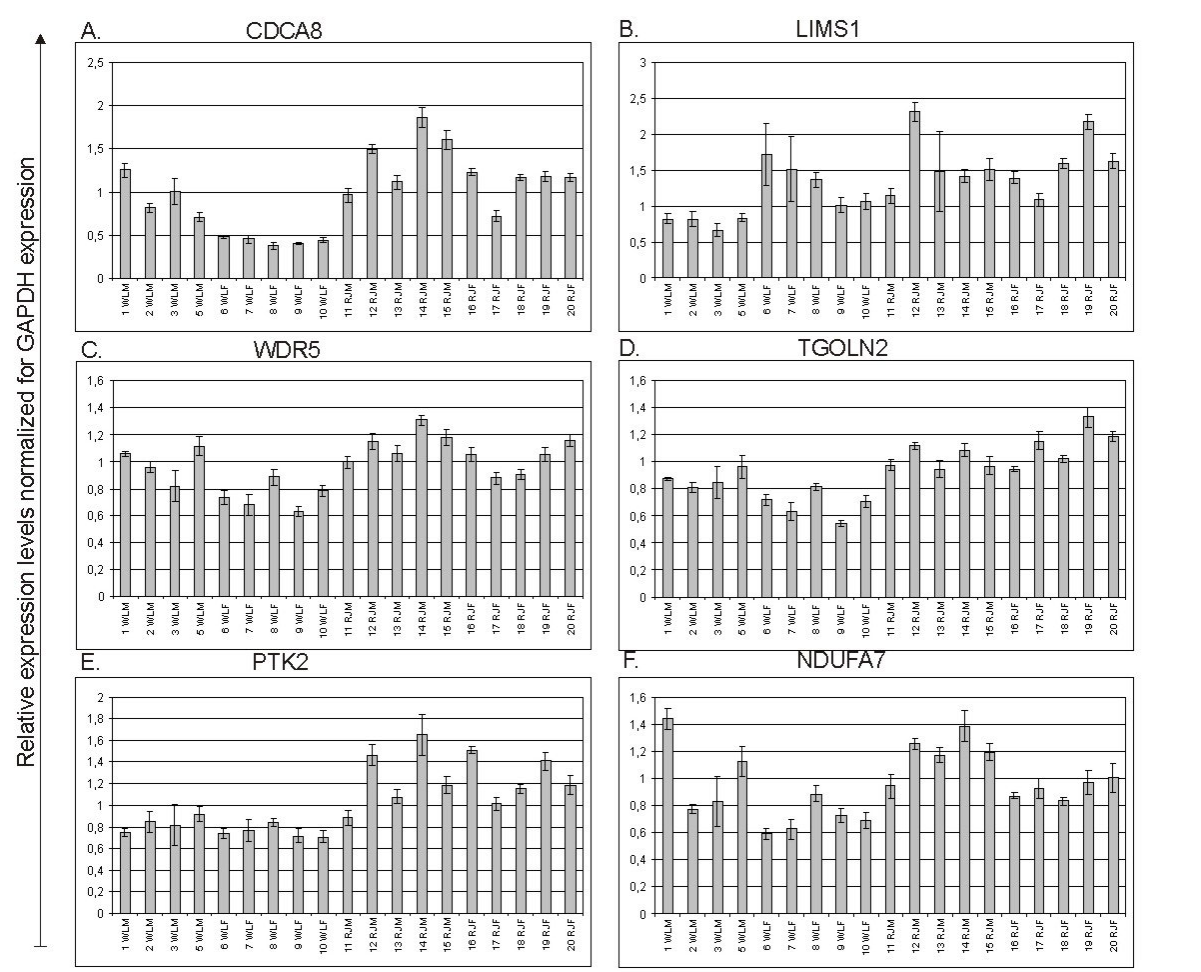
Results derived from quantitative PCR analysis of six transcripts expressed higher levels in the red junglefowl in the microarray analysis. Chicken individuals are presented along the x-axis: WLM = White Leghorn male, WLF = White Leghorn female, RJM = red junglefowl male, RJF = red junglefowl female. For each transcript, expression relative to GAPDH-expression is presented on the y-axis. Error bars indicate standard deviations.

**Figure 5:**
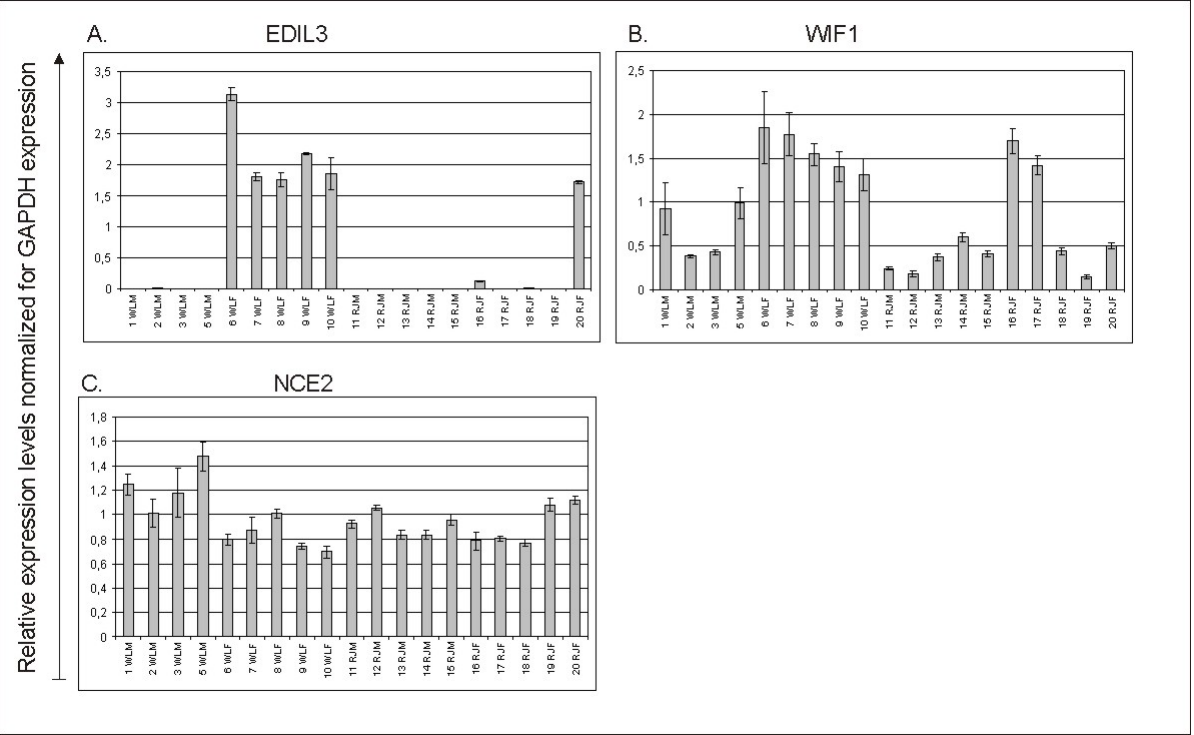
Results derived from quantitative PCR analysis of three transcripts expressed higher levels in the White Leghorn in the microarray analysis. Chicken individuals are presented along the x-axis: WLM = White Leghorn male, WLF = White Leghorn female, RJM = red junglefowl male, RJF = red junglefowl female. For each transcript, expression relative to GAPDH-expression is presented on the y-axis. Error bars indicate standard deviations.

**Figure 6:**
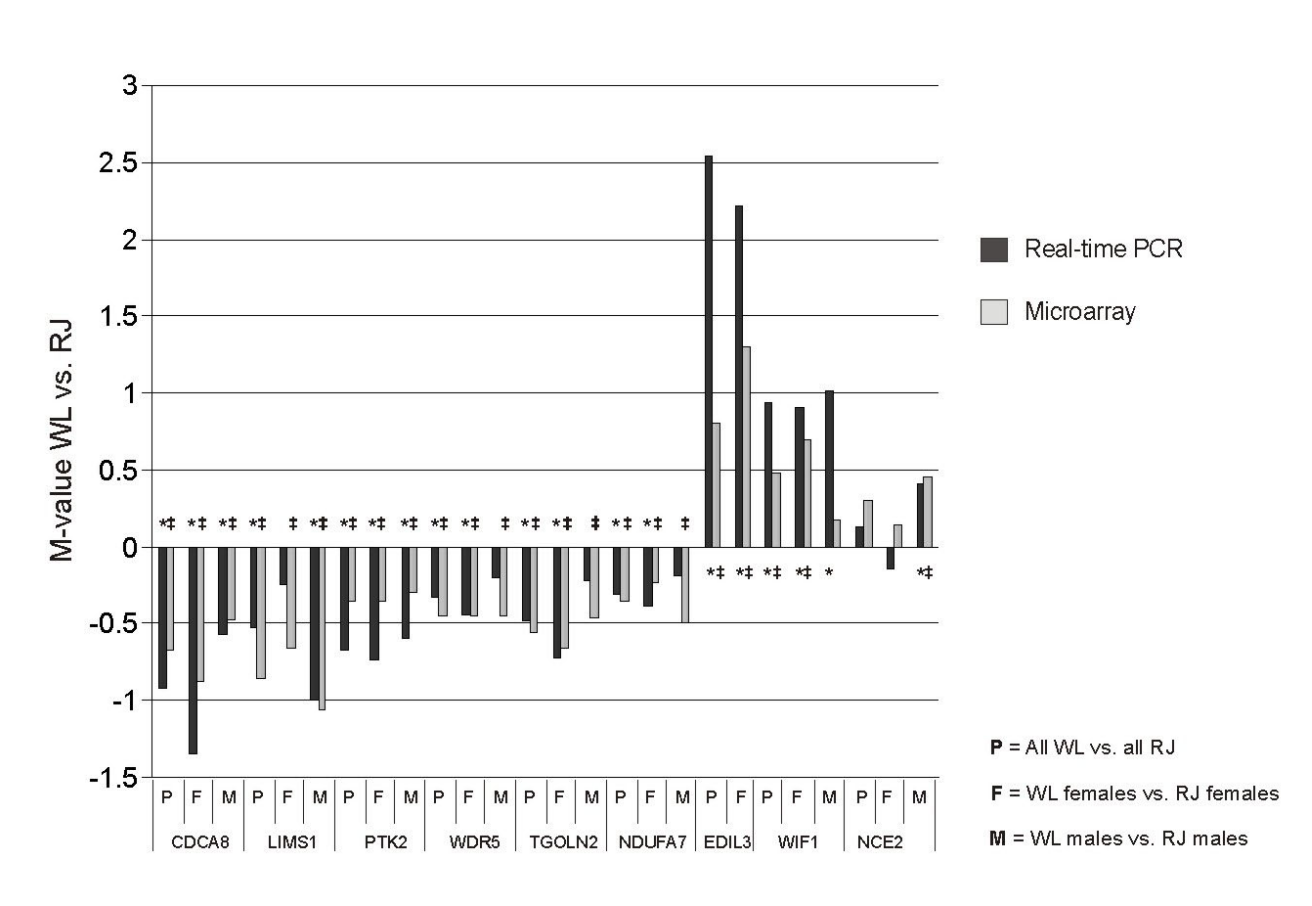
Comparison of results obtained by microarray-analysis and by qPCR-analysis. On y-axis, positive M-values indicate more expression in WL, whereas negative M-values indicate more expression in RJ. Nine genes included in qPCR-analysis are presented along x-axis. To indicate statistical significance in real-time PCR at the p < 0.05 level the * symbol is used. To indicate q-values < 0.015 in the microarray analysis the ‡ symbol is used.

Nine genes were chosen for verification of DE by quantitative PCR (qPCR). These nine genes were identified as DE between WL and RJ in the microarray analysis, some of them with high statistical significance for DE while others had q-values only slightly below 0.015. Data from qPCR revealed statistically significant DE (P < 0.05 in Student's t-test) between WL and RJ for all nine transcripts. Results from qPCR-analysis are presented in Figure 4 and in Figure 5. In Figure 6, M-values observed in qPCR-analysis are presented together with corresponding values from the microarray analysis.

### Differential expression in QTL-regions for femoral bone traits

**Figure 7:**
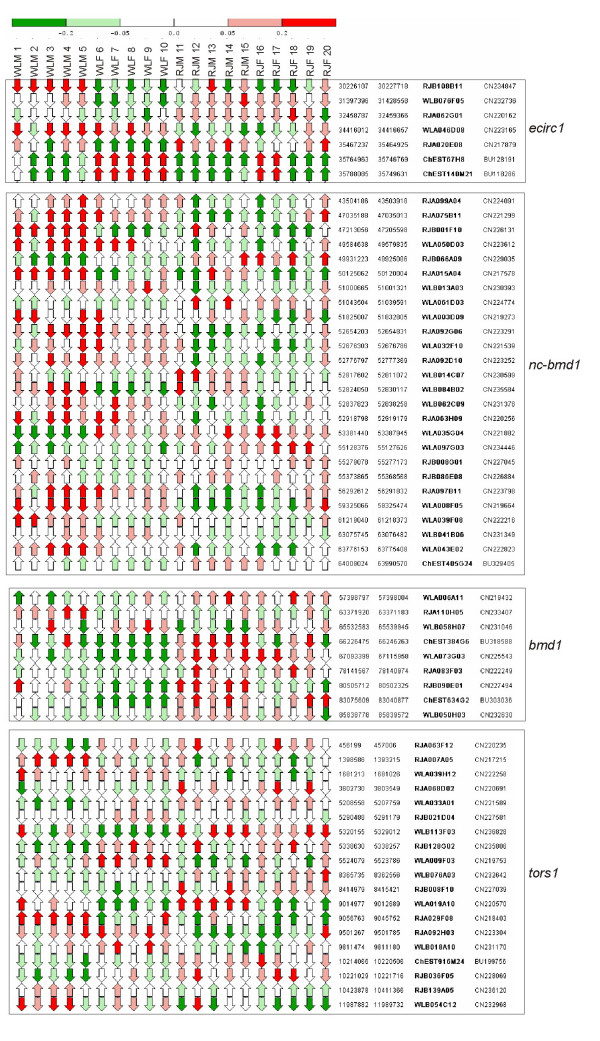
Relative expression values of differentially expressed transcripts located in QTL-regions. Along each QTL-interval, directions of arrows visualize orientation of expression (i.e. sense or antisense strand). Arrow colors visualize direction and magnitude of log2(sample channel fluorescence/reference channel fluorescence) according to upper scale bar. Red arrows indicate more expression in sample than in reference whereas green arrows indicate more expression in reference.

In total 916 probes on the microarray were present in the four QTL-regions (*ecirc1 *= 147 probes, *nc-bmd1 *= 354 probes, *bmd1 *= 148 probes and *tors1 *= 267 probes). Of 837 probes showing DE between WL and RJ, 60 were localized to QTL-regions (Table 3) and these correspond to 57 unique transcripts. In Figure 7, relative expression of the 60 DE probes is graphically presented along each QTL-interval. Of 13,907 probes spotted on the microarray, 6.6% were present within QTL regions. Out of 837 probes showing DE, 7.2% were localized to QTL-regions. Thus, there was no apparent overrepresentation of DE in QTL-regions.

**Table 3:** Differentially expressed transcripts located to QTL-regions for bone traits

Genbank ID	Pos.^1^	Ensembl gene^2^	Annotation ^3^	QTL^4^	DE ^5^	M-values^6^
[GenBank:CN234847]	1:30.2	ENSGALG00000009494	Putative orthologue to human THAP domain containing 5 (THAP5)	ecirc1	M	0.36 -0.02 0.18
[GenBank:CN232736]	1:31.3	ENSGALG00000009547	Putative orthologue to human periphilin 1 (PPHLN1)	ecirc1	F P	-0.09 -0.29 -0.19
[GenBank:CN220162]	1:32.4		NA	ecirc1	P	-0.17 -0.19 -0.18
[GenBank:CN223165]	1:34.4 ±	ENSGALG00000009762	Gag polyprotein	ecirc1	M P	0.49 0.32 0.40
[GenBank:CN217879]	1:35.5	ENSGALG00000009829	Putative orthologue to Ribosomal protein L18 A (RPL18A)	ecirc1	P	-0.20 -0.34 -0.27
[GenBank:BU128191]	1:35.7	ENSGALG00000009867	Wnt inhibitory factor 1 (WIF1) *	ecirc1	F	0.17 0.68 0.43
[GenBank:BU118286]	1:35.7	ENSGALG00000009867	Wnt inhibitory factor 1 (WIF1) *	ecirc1	F	0.15 0.64 0.39
[GenBank:CN224091]	1:43.5	ENSGALG00000010992	Putative orthologue to Solute carrier family 6, member 15 (SLC6A15)	nc-bmd1	P	0.26 0.14 0.20
[GenBank:CN221299]	1:47.0		NA	nc-bmd1	M P	0.41 0.21 0.31
[GenBank:CN226131]	1:47.2	ENSGALG00000011325	NADH dehydrogenase [ubiquinone] 1 alpha subcomplex subunit 12 (NDUFA12)	nc-bmd1	M	0.30 -0.00 0.14
[GenBank:CN223612]	1:49.5	ENSGALG00000011651	Putative orthologue to ADP-ribosylation factor-like 1 (ARL1)	nc-bmd1	P	0.36 0.35 0.36
[GenBank:CN229035]	1:49.9	ENSGALG00000023235	NA	nc-bmd1	M P	-0.33 -0.27 -0.30
[GenBank:CN217578]	1:50.1	ENSGALG00000011798	Putative orthologue to heme-binding protein1 (HEBP1)	nc-bmd1	M	0.71 0.42 0.55
[GenBank:CN230393]	1:51.0	ENSGALG00000011856	NA	nc-bmd1	P	0.16 0.14 0.15
[GenBank:CN224774]	1:51.0	ENSGALG00000011859	Eye-globin [UniProt:Q5QRU6]	nc-bmd1	P	-0.20 -0.20 -0.20
[GenBank:CN219273]	1:51.8	ENSGALG00000012013	Putative orthologue to Solute carrier family 25, member 17 (SLC25A17)	nc-bmd1	F	0.08 0.31 0.20
[GenBank:CN223291]	1:52.6		NA	nc-bmd1	M P	0.48 0.26 0.37
[GenBank:CN221539]	1:52.6		NA	nc-bmd1	P	0.21 0.30 0.26
[GenBank:CN223252]	1:52.7	ENSGALG00000019317	Putative orthologue to Josephin domain-containing 1 (JOSD1)	nc-bmd1	M P	0.29 0.17 0.23
[GenBank:CN230599]	1:52.8		NA	nc-bmd1	M	-0.16 0.00 -0.08
[GenBank:CN235584]	1:52.8	ENSGALG00000012247	Putative homologue to DEAD/H BOX 17 (DDX17)	nc-bmd1	F	0.09 -0.30 -0.10
[GenBank:CN231378]	1:52.8		NA	nc-bmd1	P	0.15 0.13 0.14
[GenBank:CN220256]	1:52.9	ENSGALG00000012272	NA	nc-bmd1	P	0.38 0.36 0.37
[GenBank:CN221882]	1:53.3	ENSGALG00000012456	Putative orthologue to Ras-related C3 botulinum toxin substrate 2 (RAC2)	nc-bmd1	M	-0.52 -0.15 -0.34
[GenBank:CN234446]	1:55.1		NA	nc-bmd1	F P	-0.20 -0.32 -0.26
[GenBank:CN227045]	1:55.3	ENSGALG00000012569	Putative orthologue to F-box only protein 7 (FBXO7)	nc-bmd1	F	-0.00 -0.13 -0.07
[GenBank:CN226884]	1:55.3	ENSGALG00000012619	NA	nc-bmd1	F	-0.02 -0.12 -0.07
[GenBank:CN223798]	1:56.2		NA	nc-bmd1	M P	0.42 0.27 0.35
[GenBank:CN219664]	1:59.3		NA	nc-bmd1	M P	0.41 0.23 0.32
[GenBank:CN222216]	1:61.2		NA	nc-bmd1	M	0.18 -0.05 0.06
[GenBank:CN231349]	1:63.0		NA	nc-bmd1	P	0.11 0.13 0.12
[GenBank:CN222823]	1:63.7		NA	nc-bmd1	P	0.31 0.22 0.26
[GenBank:BU329405]	1:63.9	ENSGALG00000013039	BH3-interacting domain death agonist (BID)	nc-bmd1	M P	-0.14 -0.11 -0.12
[GenBank:CN219432]	2:57.4		NA	bmd1	M P	-0.25 -0.19 -0.22
[GenBank:CN233407]	2:63.3		NA	bmd1	M	0.24 0.02 0.13
[GenBank:CN231046]	2:65.5	ENSGALG00000012784	Putative orthologue to Thioredoxin domain-containing protein 5 (TXNDC5)	bmd1	M	0.29 0.09 0.19
[GenBank:BU318588]	2:66.2	ENSGALG00000012802	Putative orthologue to human Coagulation factor XIII A chain precursor (F13A1)	bmd1	P	-0.44 -0.46 -0.46
[GenBank:CN225543]	2:67.0	ENSGALG00000012809	Peroxisomal D3,D2-enoyl-CoA isomerase (PECI)	bmd1	ALL	-0.42 -0.42 -0.42
[GenBank:CN222249]	2:78.1	ENSGALG00000012965	Putative orthologue to family with sequence similarity 105, member A (FAM105A)	bmd1	P	-0.14 -0.17 -0.15
[GenBank:CN227494]	2:80.5	ENSGALG00000013041	Putative orthologue to T-complex protein 1 subunit epsilon (CCT5)	bmd1	M	-0.27 -0.06 -0.16
[GenBank:BU303036]	2:83.0	ENSGALG00000013094	Dopa decarboxylase (DDC)	bmd1	ALL	-0.26 -0.40 -0.33
[GenBank:CN232630]	2:85.8		NA	bmd1	M	-0.21 -0.01 -0.11
[GenBank:CN217215]	20:1.4	ENSGALG00000001903	Putative orthologue to microtubule-associated protein 1, light chain 3 alpha (MAP1LC3A)	tors	M	0.34 0.07 0.20
[GenBank:CN222258]	20:1.7		NA	tors	P	0.18 0.22 0.20
[GenBank:CN220691]	20:3.8		NA	tors	F P	-0.15 -0.19 -0.17
[GenBank:CN221589]	20:5.2	ENSGALG00000004143	Putative orthologue to tyrosine 3-monooxygenase/tryptophan 5-monooxygenase activation protein, beta isoform (YWHAB)	tors	M P	-0.23 -0.06 -0.15
[GenBank:CN227581]	20:5.3	ENSGALG00000023846	NA	tors	F	0.01 0.16 0.09
[GenBank:CN236828]	20:5.3	ENSGALG00000004170	Adenosine deaminase (ADA)	tors	F P	-0.27 -0.69 -0.48
[GenBank:CN235806]	20:5.3		NA	tors	M	-0.26 -0.09 -0.18
[GenBank:CN219753]	20:5.5		NA	tors	ALL	0.21 0.28 0.25
[GenBank:CN232642]	20:8.4	ENSGALG00000005635	Transcription factor-like 5 (TCFL5)	tors	P	-0.12 -0.11 -0.11
[GenBank:CN227039]	20:8.4	ENSGALG00000005652	NA	tors	P	-0.12 -0.22 -0.17
[GenBank:CN220570]	20:9.0	ENSGALG00000005849	Putative orthologue to chromosome 20 open reading frame 149 (C20orf149)	tors	M	-0.38 0.01 -0.18
[GenBank:CN218403]	20:9.0	ENSGALG00000005887		tors	M P	0.52 0.26 0.39
[GenBank:CN223304]	20:9.5	ENSGALG00000006046	Putative orthologue to DnaJ homolog subfamily Cmember 5 (DNAJC5)	tors	M P	0.37 0.29 0.33
[GenBank:CN231170]	20:9.8		NA	tors	P	0.15 0.23 0.19
[GenBank:BU199756]	20:10.2	ENSGALG00000006657	Microtubule-associated protein RP/EB family member 1 (MAPRE1) *	tors	M P	-0.20 -0.13 -0.16
[GenBank:CN228069]	20:10.2	ENSGALG00000006657	Microtubule-associated protein RP/EB family member 1 (MAPRE1) *	tors	M P	-0.35 -0.18 -0.26
[GenBank:CN236120]	20:10.4	ENSGALG00000006817	Putative orthologue to catenin, beta like 1 (CTNNBL1)	tors	P	0.08 0.10 0.09
[GenBank:CN232968]	20:11.9	ENSGALG00000007768	Putative orthologue to Aurora kinase A (AURKA)	tors	P	0.34 0.43 0.39

* WIF1 and MAPRE1 are represented by two clones on the microarray± [GenBank:CN223165] may represent a retroviral element and may be present also elsewhere in the genome

1 Chromosome and positions (in Megabases) of transcripts are based on build 2.1 of the chicken genome

2 Where applicable, Ensembl gene from which transcripts are derived

3 Gene annotation. If applicable, gene name or name of putative orthologue are presented. NA = Not applicable, due to lacking annotation.

4 Previously identified QTLs for traits of femoral bone. ecirc1 = sex independent QTL for endosteal circumference, nc-bmd1 = female specific QTL for noncortical bone mineral density (BMD), bmd1 = female specific QTL for various measurements of BMD, tors1 = female specific QTL affecting femoral elasticity in torsion test.

5 The comparison between WL and RJ in which transcript was found differentially expressed (FDR-adjusted p-value < 0.0015. M = males, F = females, P = population (all WL compared to all RJ), ALL = M, F and P.

6 M-values log2(fold change) for M, F and P-comparison, respectively. Negative M-values indicate lower expression in WL.

### Gene ontology pathway analysis

GO-analyses of DE-transcripts revealed statistically significant overrepresentation of functional classes belonging to all three top-level ontologies (cellular component, molecular function and biological process). Transcripts encoding ribosomal proteins were highly enriched in the ontology "Cellular Component" and in the ontology "Molecular Function", protein biosynthesis was similarly overrepresented. Table 4 lists GO-terms overrepresented among transcripts identified as DE between RJ and WL. GO-categories overrepresented among transcripts identified as DE between males and females is presented in Additional File 7.

**Table 4:** Overrepresented Gene Ontologies among 604 probes showing differential expression in sex-independent comparison between WL and RJ.

Type ^1^	GO-ID ^2^	GO-Name	List Hits ^3^	Total Hits ^4^	EASE-score ^5^
C	GO:0005842	cytosolic large ribosomal subunit (sensu Eukaryota)	12	45	6.21 E-09
P	GO:0006412	protein biosynthesis	16	143	1.88 E-07
C	GO:0005843	cytosolic small ribosomal subunit (sensu Eukaryota)	8	32	3.53 E-06
C	GO:0005840	ribosome	9	45	6.91 E-06
P	GO:0007165	signal transduction	24	428	6.08 E-05
F	GO:0003735	structural constituent of ribosome	11	97	1.79 E-04
P	GO:0000004	biological_process unknown	72	2067	2.28 E-04
F	GO:0003723	RNA binding	20	268	2.41 E-04
P	GO:0006461	protein complex assembly	7	62	4.98 E-04
C	GO:0005856	cytoskeleton	11	108	5.03 E-04
P	GO:0006508	proteolysis and peptidolysis	10	131	8.84 E-04
C	GO:0005813	centrosome	6	39	1.05 E-03
C	GO:0005794	Golgi apparatus	9	94	2.45 E-03
P	GO:0006955	immune response	7	81	2.50 E-03
P	GO:0007268	synaptic transmission	9	128	2.78 E-03
P	GO:0006629	lipid metabolism	6	64	3.29 E-03
F	GO:0005554	molecular_function unknown	62	1461	5.76 E-03
F	GO:0016301	kinase activity	6	57	7.39 E-03
F	GO:0008248	pre-mRNA splicing factor activity	6	58	8.06 E-03
C	GO:0005622	intracellular	12	178	9.61 E-03
C	GO:0008372	cellular_component unknown	71	1763	0.013
P	GO:0006468	protein amino acid phosphorylation	9	171	0.017
P	GO:0008283	cell proliferation	7	134	0.035
C	GO:0005634	nucleus	62	1587	0.035
F	GO:0003924	GTPase activity	6	81	0.037
C	GO:0016021	integral to membrane	47	1179	0.047

1 Top-level Gene Ontology: P = biological process, C = cellular component, F = molecular function

2 Gene Ontology IDs of enriched terms

3 List Hits to Category: Numbers of DE probes on microarray belonging to specific GO-IDs. Total numbers of occurrences of C, P and F among 604 DE probes were: C = 408, P = 403 and F = 415.

4 Total Hits to Category: Total numbers of probes on microarray belonging to specific GO-ID. Total numbers of occurrences of C, P and F among all probes on microarray were: C = 13074, P = 17435 and F = 13433.

5 EASE-Score: Indicates the statistical significance of the observed overrepresentation.

### Phenotyping of the distal femoral metaphysis by pQCT

Mean noncortical BMD (BMD of trabecular and medullary bone) was 282 ± 49 mg/cm^3 ^for RJ females and 348 ± 27 mg/cm^3 ^for WL females (Additional File 4). Male pQCT images are presented in Additional File 5.

## Discussion

To our knowledge, very few studies have compared global gene expression between domestic animals and their wild ancestors [15]. Numerous studies have however examined differences in global gene expression between humans and other primates [16-22] as well as between selection lines of various species [23-28]. In the field of reverse genetics, gene expression profiling has been extensively utilized for functional inference of individual genes. In the bone research field, gene expression microarrays have been restricted mainly to studies of genetically altered mice, pharmacological studies *in vivo *and *in vitro *and transfection experiments in cell-lines.

Comparative genomic and transcriptomic studies of domestic animal species can provide insights into molecular pathways which have been perturbed through selection and can ultimately lead to the identification of genes or even mutations having been selected for during the establishment of desired phenotypes in domestic lines [29]. Having chosen the path of comparative transcriptomics, experimental design is a crucial parameter, as environmental factors may otherwise be responsible for observed differences in gene expression. Comparing gene expression of wild animals captured in their natural habitat to their domestic counterparts will result in an unknown extent of differential expression due to differing environmental factors. In the present study, all chickens were hatched, housed and sacrificed in the same facility and were thus exposed to the same environment (including feed, temperature, light schedule, pathogens, etc.) throughout life. All individuals were sacrificed at 40 weeks of age which corresponds to the age of peak bone mass [30]. At peak bone mass WL have mechanically stronger and denser bones than RJ, while later in their lives a large proportion of WL-hens develop osteoporosis due to their extremely high egg production. It should be noted that the two chicken lines differ in the age at which they enter sexual maturity (approximately 20 weeks of age for WL and 25 weeks of age for RJ) [31]. The difference between strains with regard to time since sexual maturity could be a confounding factor, as e.g. hormonal profiles may differ between the strains. Nevertheless, at 40 weeks of age chickens should be sexually mature, although age related decline in bone mass should not yet have been initialized.

When comparing gene expression in bone from two populations as divergent as RJ and WL, some confounding factors are difficult to eliminate. Although specific care was taken to eliminate confounding environmental factors when breeding the chickens, several remain and should be acknowledged. Such factors include differing ages at which the two chicken lines enter sexual maturity, potential relative differences in cell type abundance between lines as well as not having documented female egg-laying parameters before tissue sampling. Furthermore, the large difference in body size as well as bone size between adult RJ and WL constitute a potential limitation as it could lead to relative differences in cell type abundance and also differences in load applied to bones. Comparing female femurs is particularly liable to confounding factors introduced by phenotypic differences, as amounts of medullary bone can be heterogeneous. It would be optimal to compare gene expression between the two populations also at other ages, as this could help separate genes whose expression is affected factors such as load and relative differences in cell type abundance from those whose differential expression is caused by certain alleles having been fixed during domestication or evolution. Another potential bias lies in the probe composition of the microarray. The majority of the 13907 probes spotted on the microarray were derived from RJ and WL brain- and testis Expressed Sequence Tag (EST) libraries (n = 12742), but an additional 1136 clones were specifically chosen because of their biological functions. It is possible that some genes important to bone metabolism, in particular ones with expression limited to certain cell types or those expressed in low levels, may not be represented on the microarray. Despite this potential bias in probe composition, the microarray covers a large fraction of chicken transcripts and should enable global expression profiling. It may however be worthwhile to replicate these studies utilizing commercially available oligonucleotide microarrays, which would enable expression profiling of a more complete set of chicken genes. In this study five biological replicates from each sex and strain were used, rendering a total of twenty samples. It is conceivable that analysis of a larger sample size would have enabled the detection of additional genes as differentially expressed between RJ and WL. Furthermore, in order to infer with certainty gene expression signatures associated to differences in complex phenotypes such as BMD, significantly larger sample sizes than those used herein accompanied by thorough phenotype data would be required.

### Differential expression between males and females

Comparisons between males and females rendered the highest numbers of differentially expressed transcripts (Table 1). As expected, all females and no males expressed the female specific W-chromosome gene WPKCI, a gene believed to be involved in female sex determination in birds [32]. In male birds, the femoral midshaft consists of an inner blood filled marrow cavity surrounded by an outer lining of cortical bone. The marrow cavity of the sexually mature female bird is, in addition to blood and air, also occupied by varying amounts of medullary bone lining the endosteal surfaces of cortical bone. Consequently, the female femur contains a tissue which is absent from the male femur and it can therefore be expected that a large proportion of DE observed between sexes is dependent on relative differences in cell type abundance. Another fraction of DE transcripts is likely to be directly or indirectly caused by factors not attributed to phenotypic differences, but rather to karyotype as it was recently shown that dosage compensation of the avian Z is not as effective as mammalian dosage compensation [33] (in birds males have two Z-chromosomes, whereas females have one Z- and one W-chromosome). GO-categories identified as overrepresented among transcripts which exhibit DE between sexes (Additional File 7) could consequently harbor functional categories associated to cell types present in varying ratios in female and male bones. Males and females had statistically significant differences in expression of certain known bone cell type markers. Alkaline Phosphatase (*ALP*) was expressed in higher levels in the female (M-value = 0.63) (indicating higher numbers of osteoblasts in the female bone). Phosphate-regulating Endopeptidase Homolog, X-linked (*PHEX*) also had higher expression in female bone (M-value = 0.64), which similarly would indicate higher osteoblastic numbers in female bone. The osteoclast marker Creatine Kinase B (*CKB*) was expressed in more than four times higher levels in female femurs than in male femurs. The higher expression of markers for both osteoblasts and osteoclasts suggests that bone remodeling is accelerated in female bone, and is likely attributed to females having higher turnover rates of bone tissue.

### Differential expression between RJ and WL

For nine selected transcripts, DE observed in the microarray analysis was verified by qPCR-analysis (Figure 6), indicating true differential expression for most probes having been identified as DE. As could be expected, the magnitude of DE varied between the two methods, of which qPCR is likely to be more accurate due to having greater specificity than hybridization techniques.

Higher numbers of microarray probes showing DE were observed in contrast between males of the two strains (n = 410) than in the corresponding female contrast (n = 270). In reproductive female chicken the cyclical process of egg-laying is accompanied by a phase of intense bone remodeling. It is possible that the females were in different phases of bone remodeling at the time of tissue sampling and/or that female femurs had differing relative amounts of cortical or medullary bone, which could also be calcified to different degrees. Differences in bone remodeling due to egg-laying and differences caused by heterogeneous bone phenotypes could both result in female biological replicates being more heterogeneous than male ones, and could explain why less DE was seen in the female contrast. In Figure 1, it can be seen that the fraction of DE transcripts which overlap between the male specific- and the sex independent contrasts is higher than the corresponding overlapping fraction observed between the female- and sex-independent contrasts. We suggest that this can be attributed to a larger phenotypic heterogeneity among female than male biological replicates.

Phenotyping of the femurs revealed that WL females generally had slightly higher density of noncortical BMD (in the female metaphysis medullary- and bone trabecular together constitute what here is referred to as noncortical bone) in the metaphysis with mean values of 348 ± 27 mg/cm^3 ^for WL females and 282 ± 49 mg/cm^3 ^for RJ females (Additional File 4). From phenotyping performed by pQCT in an independent sample of female chicken we have observed that noncortical BMD of the femoral metaphysis is strongly positively correlated with medullary BMD of the femoral midshaft (r = 0.73) (Additional File 6). The strain differences in noncortical BMD of the metaphysis are not large enough to conclude that they can be attributed also to the femoral midshaft. Interestingly, the metaphysis of RJ female individual 19 (RJF19) had very little, if any medullary bone (Additional File 4) and consequently a low noncortical BMD. The absence of medullary bone from RJF19 is likely to confer absence of this bone type also in the midshaft. Exclusion of RJF19 from the female RJ vs. WL contrast did not significantly alter the set of probes for which DE was observed, nor did the exclusion result in any dramatic changes in the statistical significance of observed DE (data not presented). The femoral phenotyping was performed in the metaphysis whereas the RNA was prepared from the midshaft and the data should thus be regarded as a qualitative rather than quantitative addition to the study. pQCT-data obtained for the female femurs indicate large differences in bone size between the RJ and WL populations, but also indicate that female biological replicates should be relatively homogenous with the exception of RJF19. From the phenotyping of male femurs (Additional File 5), we draw no conclusion other than that there is an apparent size difference between the two populations.

No commonly used bone cell markers were identified as differentially expressed between RJ and WL in the sex-independent contrast, nor in the two sex-specific contrasts. However, some genes for which differential expression was observed have previously been attributed roles in bone metabolism. For example WD-repeat containing protein 5 (WDR5), which accelerates osteoblast and chondrocyte differentiation [34,35] and whose expression is induced by bone morphogenic protein 2 (BMP2). Figure 4C and Figure 6 show that WL-individuals expressed *WDR5 *in lower amounts than did RJ-individuals. Over the last few years, the wnt-signaling system has been attributed roles important to bone metabolism (reviewed in [36]), and has become one of the most extensively studied signaling systems in the bone field. Wnt inhibitory factor 1 (*WIF1*) was identified as DE between females of the two strains, with WL having higher levels than RJ. WIF1 is a secreted protein that inhibits some wnt-proteins from binding to their receptors in a manner distinct from that of the secreted frizzled related protein receptors (SFRPs) [37]. Interestingly, *WIF1 *is located in a RJ/WL QTL-region originally identified for body weight and growth [38], which also has pleiotropic effects on many bone traits [11] as well as several other phenotypes [39,40]. WL females were found to have stable mRNA levels of *WIF1 *whereas expression was heterogeneous in RJ-females (Figure 5B). Strong expressional regulation of *WIF1 *has been observed during BMP2 induced osteoblastic differentiation of murine C2C12 and MC3T3 cells [41,42]. The latter study reported that treatment of C2C12-cells with LiCl had a stimulatory effect on WIF1 expression, indicating that WIF1 expression is regulated by wnt-signaling components. Furthermore, co-expression of WIF1 and the transcription factor RUNX2 in osteoblastic regions of all bones in the head and the axial skeleton was observed in mice at embryonic day 16.5 [41]. RUNX2 is a master regulator of osteoblastic differentiation, and co-expression between WIF1 and RUNX2 therefore indicates a role of WIF1 in this process. Furthermore, *WIF1 *has been reported to be expressed in trabecular but not cortical bone of mature mice [42]. This is intriguing because trabecular bone has a much faster turnover as compared to cortical bone, a relationship analogous to the contrasting lability between medullary- and cortical bone in chicken. Syndecan 3 (*SDC3*) has also previously been implicated in bone signaling [43]. It had higher expression in WL and its expression pattern was highly correlated with *WIF1 *in hierarchical clustering of all probes representing the 779 transcripts identified as differentially expressed between WL and RJ (Additional File 2). We speculate that in reproductive female chicken, expression of *WIF1 *and SDC3 is induced in medullary bone in response to bone remodeling accompanying egg-laying.

Two probes targeting immunoglobulin-like receptor *CHIR-A *([GenBank:CN233569] and [GenBank:CN233552]) indicated lower levels of expression in WL than in RJ (M-value = -1). The CHIR-A protein shows homology to osteoclast associated receptor (OSCAR), which has been shown to inhibit the formation of osteoclasts from bone-marrow precursor cells stimulated by osteoblasts [44]. Although *CHIR-A *has yet not been anchored to a chicken chromosome, synteny between human 19q13.4 and chicken micro-chromosome E64 [45] suggests location on this micro-chromosome.

LIM and senescent cell antigen-like domains 1 (*LIMS1*) and Ran binding protein 2 (*RANBP2*) are closely located on chicken chromosome 1 and were both expressed in higher amounts in RJ (Table 2). LIMS1 is a focal adhesion protein which together with integrin linked kinase 1 (ILK1) and alpha-parvin (PARVA) forms a complex which regulates cell shape, motility, and survival [46]. Also associated to focal adhesions, cytoplasmic protein tyrosine kinase (*PTK2*) and vinculin (*VCL*) were expressed in higher amounts in RJ (Table 2). PTK2 is activated by binding of ligands such as integrins and growth factors to their receptors and it was recently proposed that interactions between vinculin, talin, and actin filaments constitute a slippage interface between the cytoskeleton and integrins [47]. Large inter-individual differences in expression levels were observed for EGF-like repeats- and discoidin I-like domains-containing protein 3 (*EDIL3*). qPCR revealed that all female WL and one female RJ had at least 95-fold higher expression levels of *EDIL3 *than the other individuals most of which had no detectable expression (Fig. 3G). The EDIL3 protein adheres to endothelial cells through integrin A5/integrin B3 receptor binding [48,49] and promotes angiogenesis by inducing expression of pro-angiogenic molecules [50]. Possibly, a stable expression of *EDIL3*, as seen in WL-females is a result of them having a high turnover rate of the vascular medullary bone due to intense selection for egg production. The individual heterogeneity in expression among RJ-females could similarly be explained by their lower rate of egg production, with medullary bone not being subject to constant remodeling.

The observation that several DE transcripts have functional convergence at integrin signaling/focal adhesion is interesting and makes it tempting to speculate that these signaling pathways have become perturbed during domestication or due to selection for egg production in WL, possibly resulting in altered bone metabolism.

### Differential expression in QTL-regions

Of 779 unique DE transcripts, 57 were found to be localized within previously identified QTL-regions for bone traits (Table 3). Three of these QTLs had sex dependent effects in the RJ/WL-intercross population previously studied [11]. QTL *ecirc1 *on chromosome 1 had sex independent effects and the WL-allele conferred larger circumference of the femoral endosteum. At QTL *nc-bmd1 *also on chromosome 1, female RJ-allele homozygotes were bestowed with higher noncortical BMD (medullary and/or trabecular BMD), whereas at QTL *bmd1 *on chromosome 2 the WL-allele was associated with higher noncortical BMD as well as higher total BMD in females. For *tors1 *on chromosome 20, female WL-allele bearers have more rigid femurs in rigidity tests by torsion. In Table 3 is indicated for which WL/RJ-contrast DE was observed, as this may aid in interpreting the significance of an overlap between DE and QTL.

Peroxisomal D3, D2-enoyl-CoA isomerase (*PECI*) which is present in QTL-region *bmd1 *was expressed in higher levels in RJ (tables 1 and 2). PECI is a peroxisomal enzyme catalyzing an isomerization step required for the beta-oxidation of unsaturated fatty acids, and has not previously been implicated in bone signaling pathways. The genomic location of *PECI *is very close to the peak of QTL *bmd1 *[11] and the statistical significance as well as magnitude of differential expression (sex-independent M-value of -0.42) is similar regardless of contrast between WL and RJ. Due to these observations and despite no prior association to bone metabolism, we regard PECI as a QTL-candidate gene. Ribosomal protein L18A (*RPL18A*) was expressed in higher levels in RJ (M = -0.27) and the gene resides within QTL-region *ecirc1*. In GO-enrichment analysis, transcripts encoding ribosomal proteins were identified as overrepresented among DE transcripts, making *RPL18A *a QTL-candidate gene.

The probe targeting [GenBank:CN223165] was expressed in higher levels in WL (M = 0.40 and 0.32 in male- and sex independent contrast between WL and RJ, respectively). In protein BLAST search, [GenBank:CN223165] had highest similarity to a gag/pol polyprotein from avian myeloblastosis virus and was also highly similar to an avian endogenous retroviral insertion localized within the confidence interval of the pleiotropic QTL *ecirc1*. Interestingly, several isolates of avian myeloblastosis-associated virus (MAV) and avian leukosis virus (ALV) can induce osteopetrosis in chicken and may also affect growth (reviewed in [51]). Osteopetrosis is a bone remodeling disorder characterized by an increase in bone density, and it is therefore tempting to speculate that the retroviral insertion could be causative of the pleiotropic QTL, for which the WL-allele confers higher phenotypic values both for bone and growth traits.

Theoretically, any genetic element which segregates between RJ and WL and which is localized to a QTL-region could be responsible for QTL-effects. Based on gene function as well as magnitude and statistical significance of DE, the best candidates among herein identified DE-genes include: *WIF1, PECI, RPL18A *and the retroviral insertion represented by a probe targeting [GenBank:CN223165].

### GO-term enrichment analysis

**Figure 8:**
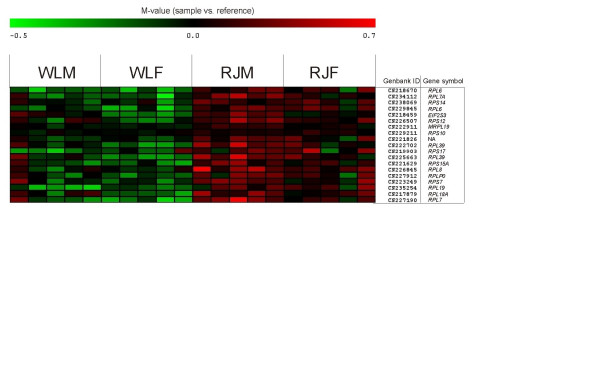
Heat map illustrating expression ratios (log2(sample fluorescence/reference fluorescence)) for probes from enriched Gene Ontology categories GO:0005842 and GO:0005843, representing cytosolic large and small large ribosomal subunits, respectively. WLM = White Leghorn males, WLF = White Leghorn females, RJM = red junglefowl males, RJF = red junglefowl females. Red colored squares indicate more expression in sample than in reference whereas green indicate more expression in reference.

GO-term enrichment-analysis of herein identified DE-probes could lead to identification of transcripts involved in signaling pathways having been perturbed between RJ and WL. It is however important to keep in mind that GO-enrichment analysis should be regarded primarily as a rough tool, which could aid in interpretation of data but also that obtained results require manual revision. The list of herein identified differentially expressed transcripts was enriched for certain GO-terms (Table 4). Prominent GO-categories identified in this study (Table 4), were those associated to the ribosome and to protein biosynthesis. Upon examination of probes contributing to overrepresentation of GO-terms; "cytosolic large and small ribosomal subunits", "protein biosynthesis", "ribosome", "structural constituent of ribosome", "RNA-binding" and "intracellular", we observed that 18 probes, representing transcripts encoding 15 ribosomal proteins were largely responsible for enrichment of these GO-terms. Inter-individual differences in expression for probes enriched in GO:0005842 and GO:0005843 are presented as a heat map in Figure 8. Probes for all 15 transcripts had lower expression levels in WL than in RJ, and had a mean M-value of -0.31. BLAST-searches of probe sequences for each ribosomal protein against all chicken cDNAs and against the chicken genome revealed localization to separate loci in the chicken genome and very low inter-sequence homology among probes, thus excluding the possibility of cross-hybridization as explanation for the observed overrepresentation. Of the eighteen probes enriched in GO:0005842 and GO:0005843, seven were derived from red junglefowl EST-libraries while the remaining eleven were derived from White Leghorn EST-libraries, suggesting that SNPs in the WL were not causative of differential expression. Interestingly, eukaryotic translation initiation factor 2, subunit 3 (*EIF2S3*) and Eukaryotic translation initiation factor 4, gamma 2 (*EIF4G2*) were also identified as DE in all contrasts between RJ and WL (Table 2) and were, analogously to the ribosomal proteins, expressed in lower levels in WL. When examining transcripts identified as DE in the sex independent contrast we noted that Eukaryotic translation elongation factor 1 alpha 1 (*EEF1A1*) and Eukaryotic translation elongation factor 2 (*EEF2*), were both expressed in lower levels in WL (M-values = -0.25 and -0.32, respectively). The observation that transcripts encoding 15 ribosomal proteins as well as several translation initiation and elongation factors had lower levels of expression in WL is intriguing. We speculate that one route, through which selection acted when desired traits were established in the WL, involved the alteration of pathways affecting protein biosynthesis.

Upon examination of transcripts contributing to other overrepresented GO-terms no apparently perturbed pathways were identified, possibly due to the limited coverage of the microarray.

## Conclusion

We have utilized microarray technology to compare global gene expression between the domestic breed White Leghorn (WL) and its wild ancestor, the red junglefowl (RJ). When contrasting gene expression between the two chicken lines we identified differential expression (DE) for 837 microarray probes, representing 779 unique transcripts. Some of the 779 DE transcripts are encoded by genes with functions with interest with regard to bone metabolism (e.g. WDR5, Syndecan 3, WIF1, CHIR-A) and 57 were found to be localized within QTL-regions for bone traits. Among DE genes located to QTL regions for bone traits, *WIF1, PECI, RPL18A *and a retroviral insertion will be pursued as candidate QTL-genes. Ongoing fine mapping of QTL-regions will decrease confidence intervals of identified QTLs and thus limit the number of DE transcripts localized therein.

In search for enriched Gene Ontology categories among DE transcripts, it was observed that transcripts encoding ribosomal proteins were highly enriched, all having lower expression levels in the WL. Further examination revealed that four transcripts encoding translation initiation and elongation factors were analogously to the ribosomal proteins also expressed in lower levels in WL, possibly suggesting that protein biosynthesis pathways have become perturbed during the selection for desired phenotypes in the WL.

## Methods

### Microarray experiments

#### Microarrays

The microarrays used in this study (KTH UniChicken 2x14k cDNAv1) were developed at the Royal Institute of Technology (RIT) in Stockholm, and comprise 13907 Expressed Sequence Tag (EST)-clones spotted in duplicate. Of the EST-clones, 12742 originated from red junglefowl and White Leghorn brain- and testis libraries manufactured at the Royal Institute of Technology in Stockholm, Sweden [52]. 1136 clones originate from the BBSRC *Gallus gallus *EST database and these were included because of their biological functions. The remaining 29 clones were created at the Department of Medical Biochemistry and Microbiology at Uppsala University by a representational difference analysis (RDA) approach. Details regarding cDNA amplification, purification and printing are available through the ArrayExpress microarray data repository using the array accession number A-MEXP-266.

#### Chicken lines, tissue sampling and RNA isolation

The red junglefowl line originates from Thailand and has been kept at a population size of approximately 30 males and 30 females for four generations. The WL line (Line L13) is kept at the Swedish University of Agriculture (SLU) and has been maintained at a population size of 30 males and 30 females. The L13-line is not a commercial hybrid line, but has been specifically selected for egg-weight since the beginning of the 1970's [53]. All animals were kept in the same facility during their entire lives (further described in [40]). Briefly, the animals were kept in groups of about 35 animals in pens measuring 3 × 3 m, containing perches, wood-shavings, commercial chicken feed and water, both ad lib. When the birds started to lay eggs, the pens were equipped with group nests on the floor. Room temperature was kept at about 22 degrees Celsius, and light levels at about 30 Lux, with a 12/12 hour light/dark rhythm. Five individuals representing each sex from WL (strain L13) and from red junglefowl were sacrificed at 40 weeks of age. Immediately *post mortem *femoral bones were thoroughly stripped of soft tissue and were then snap frozen in liquid nitrogen and stored at -70°C until further use. Midshafts of femurs were ground to a fine powder with a mortar while being submerged in liquid nitrogen. RNA was extracted with Trizol (Invitrogen) and the resulting total RNA was purified on RNeasy spin columns (Qiagen, Hilden, Germany) according to the manufacturer's instructions. Twenty μg of individual RNA samples and reference RNA samples (pool consisting of mixed purified RNA from all twenty individuals) were frozen at -70°C until hybridization experiments were initiated. These experiments were approved by the animal research ethics committee in Gothenburg, Sweden (approvals 256/99 and 147-2001).

#### Assessment of RNA integrity

All RNA samples were analyzed with the Bioanalyzer (Agilent technologies) and spectrophotometer (Nanodrop). For all samples, the Bioanalyzer data revealed distinct 28S- and 18S ribosomal RNA bands and 28S/18S ribosomal RNA ratios ranged between 1.2 and 1.5, indicating RNA of quality suitable for expression analysis.

#### cDNA-synthesis, fluorophore labeling, hybridization and washing

20 μg RNA from each individual and 20 μg reference RNA were subjected to cDNA synthesis using reverse transcriptase (Superscript II, Invitrogen) for 2 h at 42°C, during which deoxyribonucleic acids and aminoalyl-labeled Uracil were incorporated into cDNA-strands. Remaining RNA was removed from the cDNA sample by NaOH mediated hydrolysis [54] (protocol SOP 002). Purified aminoalyl-labeled cDNA was purified on silica membrane spin colons (Qiagen, Hilden, Germany), after which one of the fluorophores Cy3 or Cy5 (Amersham biosciences) were attached to the aminoalyl at Uracil residues. Two subsequent silica membrane spin column purification steps were performed to remove unbound fluorophores from cDNA.

In the following step, individual cDNA-samples were hybridized together with the differently labeled reference cDNA on the microarray. Each individual sample was subjected to two hybridizations (dye-swap hybridizations), i.e. the samples were all labeled with Cy3 in one experiment and with Cy5 in another experiment. The hybridizations were performed in five separate batches; each batch included samples from all groups of individuals i.e. (WL-females, WL-males, RJ-females and RJ-males) and also included an equal number of Cy3 and Cy5 labeled samples. Protocols for hybridization and washing were developed at KTH and can be found at the website [54] as protocol SOP 003.

#### Microarray scanning, feature identification and flagging

Microarrays were subjected to 18 h of hybridization at 42°C while kept in waterproof containers in a water bath, and were subsequently scanned with a G2565BA DNA microarray scanner (Agilent technologies, U.S.A.). Scanning was performed with photo multiplier tube (PMT) settings that gave balanced signals from the two channels. Data-files containing raw fluorescence data (TIFF-files) were imported into the software GenePix (Molecular Devices Corp., Union City, CA, U.S.A.), in which TIFF-files generated for Cy3- and Cy5-channels were superimposed upon each other. Spot identification, manual examination of the surface of the array and flagging of spots/regions with poor quality were all performed in GenePix. Files are available through the ArrayExpress experiment repository (accession number E-TABM-241).

#### Data analysis

All microarray analysis steps were conducted with the computer software R version 2.4.1 [55] with the additional KTH-package [54]

#### Filtration and normalization of data

For each array, filtration was performed on spots that had been flagged either manually or by the default settings in the GenePix software. Subsequent filtering was performed for the size of spots, background vs. foreground signal intensity, intensity ratio of fluorescence from the two channels and saturated spots. To normalize signal intensity over the surface of each individual slide and thereafter to normalize signal intensity between all slides, print-tip-lowess normalizations were applied [56]. Approximately 70% of all spots on the arrays were retained after filtration and normalization.

#### B-test

Differentially expressed genes were identified using a B-statistic available in LIMMA [57]. The "B-value" assigned to each gene is the log posterior odds ratio of differential expression versus non-differential expression [58,59]. Correlations between within-array replicate spots were integrated in the statistical model as described in [60]. In total, three contrasts between WL and RJ were performed (male vs. male, female vs. female, and all WL vs. all RJ). In each contrast, individuals belonging to the same group were treated as biological replicates. In the statistical test, probabilities for differential expression (p-values) were determined for each probe, with these p-values then being adjusted for false discovery rate (FDR) [61]. Probes having obtained FDR-adjusted p-values (q-values) < 0.015 were considered differentially expressed.

### Quantitative PCR

Individual RNA-samples were subject to DNAse treatment using TURBO-DNAfree (Ambion) and equal amounts of RNA-samples were then reversely transcribed with the High Capacity cDNA reverse transcription kit (Applied Biosystems). Sample cDNA was diluted 20-fold with nuclease-free water and a reference chicken cDNA-sample was diluted 2-, 5-, 10-, 20-, 50-, 100- and 500-fold. Ten μl 2 × TaqMan^® ^Universal PCR Master Mix, No AmpErase^® ^UNG (Applied Biosystems) was mixed with 9 μl diluted cDNA and 1 μl of Taq-man gene specific assay mix (Applied Biosystems). This mix was subjected to 40 cycles of polymerase chain reaction amplification using the ABI Prism 7900 Taqman instrument (Applied Biosystems), where the amount of fluorescence is measured after each cycle. Diluted cDNA-samples from each individual were analyzed in triplicate and each dilution of the reference cDNA was analyzed in duplicate for the purpose of standard curve generation. For all TaqMan assays, correlation coefficients of standard curves exceeded R^2 ^= 0.98 and standard curve method was subsequently used for relative quantification of target abundance. All assays were designed with the Assays-by-Design software (Applied Biosystems) and featured gene specific primers and exon-spanning reporter probes (Table 5). Glyceraldehyde 3-phosphate dehydrogenase (*GAPDH*) was used as reference gene and for each individual the relative expression level of each transcript was normalized relative to GAPDH-expression.

**Table 5:** Taq-man qPCR-assay details

**Gene name**	**Ensembl transcript ID **^1^	**GenBank ID **^2^	**Forward primer**	**Reverse primer**	**Reporter Probe**
WDR5	ENSGALT00000004189	[GenBank:CN225644]	CTCTTAAGCTTTGGGACTACAGCAA	AGTACTTCTCATTTTTGTGTCCTGTGT	TCTTCAGGCACTTTCC
PTK1	ENSGALT00000026060 ENSGALT00000026061 ENSGALT00000030798	[GenBank:CN232734]	TCCGCCAGAGGAGTACGT	GCGACTCATCCACTGTTGCTA	CAAGCCAACCTCCTTTACC
TGOLN2	ENSGALT00000023308	[GenBank:CN223571]	CGGCGGCCCAAATCTG	GCAGCTTTCTTCCACTGAATACCT	CCTGGATCAAAAGATCTAG
WIF1	ENSGALT00000016042	[GenBank:BU128191]	CGGGATTCGAAGGAGACCAAT	TCTTACCCATACATTTTCCTCCATTTCG	ATGGCATTTACTGATTTCAC
GAPDH	ENSGALT00000014280 ENSGALT00000023323	[GenBank:CN233267]	GGAGTCCACTGGTGTCTTCAC	GCTGAGATGATAACACGCTTAGCA	CCCTTCAGATGAGCCCCAG
EDIL3	ENSGALT00000025199 ENSGALT00000025200 ENSGALT00000025201	[GenBank:CN224434]	CCTACAAGCTTGCCTACAGTAATGA	ACATTCTTCCGGTGTGTGTCAT	CCTGGAAAACCTTGTCCTTC
CDCA8	ENSGALT00000000432 ENSGALT00000008916	[GenBank:CN226240]	GTCTTCATTACCGTTCCTGTTGGA	CCTCCGGATTGAGATGAAGAAGATT	CTGTTAAACGAATGCTCTCCC
LIMS1	ENSGALT00000027130	[GenBank:CN228750]	GCAGTTTCCTGAAGGGCTCTT	ACCACACTGATGGCAGCAA	TTCCTCCCTTCAAACTCAT
NCE2	ENSGALT00000006045	[GenBank:CN227630]	GCTGGGCTCCAACTAGAACATT	CAAAGGATCGTCAAAATTCAGAAGATCAG	CCCCAGACAACATCCTTT
NDUFA7	ENSGALT00000000895	[GenBank:CN225183]	CGGCTCCGCAACTTCCT	GCTGCGTCCTCTTGGAGATC	CTGGAGGTCCCGCCCC

^1 ^Transcripts targeted by each Taq-man assay. Specified are Ensembl transcript IDs.

^2 ^GenBank ID of probe for which assay was designed.

### Analysis of differential expression within QTL-regions

Chromosomal intervals were defined for our four previously identified QTLs for femoral bone traits [11], entitled *ecirc1, nc-bmd1, bmd1 *and t*ors1*. Confidence intervals (CIs) of QTLs were defined as one unit LOD-drops at both sides of peak LOD-scores. For all QTLs, CIs were delimited by centiMorgan positions located in between microsatellite markers. As a conservative measure, QTL-regions were defined as chromosomal positions confined by microsatellite markers flanking CIs. Chromosomal positions were obtained from the chicken genome draft sequence build 2.1 [62].

### Overrepresentation analysis of Gene Ontology terms

Clones on the microarray were attributed Gene Ontology (GO) terms by BLAST-analysis of all individual probe sequences against GO peptide sequences obtained from the Gene Ontology project website [63]. BLAST search hits with a similarity cut-off of greater than E = e^-10 ^were retained for use in a modified version of the EASE-software [64]. In EASE, GO-term enrichment analysis was performed for probes differentially expressed between all WL and all RJ. In the analysis, each GO-term was assigned an EASE score which is a conservative adjustment of Fisher's exact probability utilizing a jackknife approach. Enriched GO-terms (EASE-score < 0.05) comprising six or more DE probes on the microarray were retained for further analysis.

### Clustering of gene expression data

Clustering was performed in MultiExperiment Viewer (MeV) [65]. Normalized fluorescence intensity values of each dye-swapped experiment were averaged separately for sample and reference channels. Thereafter, for each probe and individual, averaged sample and reference fluorescence values were log2-transformed. Average linkage hierarchical clustering was performed using Euclidian metric. In heat-maps the color of features (probes) were determined by log2(sample/reference).

### Phenotyping of the femoral metaphysis

The femurs, from which RNA had previously been prepared from the midshaft were subjected to phenotyping by peripheral Quantitative Computerized Tomography (pQCT). pQCT was performed with the Stratec pQCT XCT Research SA instrument (Stratec Medizintechnik) operating at a resolution of 70 μm. Noncortical BMD, which in the female bird reflects BMD of both trabecular and medullary bone was determined *ex vivo*, with one metaphyseal pQCT scan of the region situated at approximately 5% of bone length from the distal end of femur, and the noncortical bone was defined by setting an inner threshold to density mode (600 mg/cm3).

## Abbreviations

cDNA: Complementary DNA

DE: Differential expression

GO: Gene Ontology

QTL: Quantitative Trait Loci

## Authors' contributions

CJR carried out the molecular genetic studies, performed statistical analyses and drafted the manuscript. JLI conceived software for GO-analysis and performed statistical analyses. CF collected tissue, was involved in array creation (cDNA libraries and RDA clones) and contributed to qPCR- and GO-analysis. PS developed the microarray. JLU contributed to experimental design and provided microarray facility resources. PJ was responsible for breeding and maintenance of chicken lines. AK and LA conceived the study and participated in its design and coordination. All authors contributed to, read and approved the final manuscript.

## Supplementary Material

Additional File 1 Top tables of differentially expressed probes (q-value < 0.015) identified in contrast All WL vs. All, RJ, and in corresponding sex-specific contrasts. Positive M-values indicate higher expression in WL, whereas negative M-values indicated more expression in RJ. Click here for file

Additional File 2 Hierarchical clustering of microarray data for 837 probes, corresponding to 779 differentially expressed in three contrasts between WL and RJ. Click here for file

Additional File 3 Along x-axis, individuals included in experiment are presented (1–5 = WL males, 6–10 = WL females, 11–15 = RJ males and 16–20 = RJ females) On the y-axis, mean M-value for sample vs. reference is presented. Mean M-values are based on signal from 124 separate spots on microarray all containing a probe targeting GAPDH (used as an endogenous control in the microarray experiment) Click here for file

Additional File 7 Overrepresented Gene Ontologies (GOs) among 3372 probes showing differential expression (q-values < 0.015) in contrast between ten males and ten females (all males compared to all females). Presented are GO-terms which obtained EASE-score < 0.05 and which comprised six or more DE probes on the microarray. Top-level Gene Ontologies are denoted: P = biological process, C = cellular component, F = molecular function. List Hits to Category indicates numbers of DE probes on microarray belonging to specific GO-IDs. Total numbers of occurrences of C, P and F among 3372 DE probes were: C = 2226, P = 2283 and F = 2224. Total Hits to Category indicates total numbers of probes on microarray belonging to specific GO-ID. Total numbers of occurrences of C, P and F among all probes on microarray were: C = 13074, P = 17435 and F = 13433. The EASE-Score indicates the statistical significance of the observed overrepresentation Click here for file

Additional File 4 Metaphyseal images of female femurs from which RNA was derived for the microarray study. Images were derived from phenotyping of the femoral metaphysis by peripheral Quantitative Computerized Tomography (pQCT). The same femoral bones, from which RNA was prepared from the midshaft, were phenotyped in the distal metaphysis by one pQCT-scan at approximately 5% of bone length. Arrows indicate appearance of trabecular bone and medullary bone as well as the absence of medullary bone from red junglefowl female number 19. The noncortical bone mineral density (BMD) in mg/cm^3 ^which in the female metaphysis represents the density of a mix between trabecular and medullary bone is presented below the images corresponding to each individual. The number corresponding to each individual is presented above the images. Click here for file

Additional File 5 Metaphyseal images of male femurs from which RNA was derived for the microarray study. Images were derived from phenotyping of the femoral metaphysis by peripheral Quantitative Computerized Tomography (pQCT). The same femoral bones, from which RNA was prepared from the midshaft, were phenotyped in the distal metaphysis by one pQCT-scan at approximately 5% of bone length. The number corresponding to each individual is presented above the images. Click here for file

Additional File 6 Correlation between noncortical BMD of the distal femoral metaphysis and medullary BMD of the femoral midshaft. Results are based on an independent sample consisting of 313 female chicken studied at 200 days of age. Phenotyping was performed by peripheral Quantitative Computerized Tomography and noncortical bone was defined by setting the inner thresholds to 600 mg/cm^3 ^and 1000 mg/cm^3 ^at the distal metaphysis and midshaft, respectively. Click here for file
