# Supplementary figures and images for: Differential gene expression in femoral bone from red junglefowl and domestic chicken, differing for bone phenotypic traits
Source: BMC Genomics. 2007 Jul 2;8:208. doi: 10.1186/1471-2164-8-208 (PMC1934367; doi:10.1186/1471-2164-8-208)

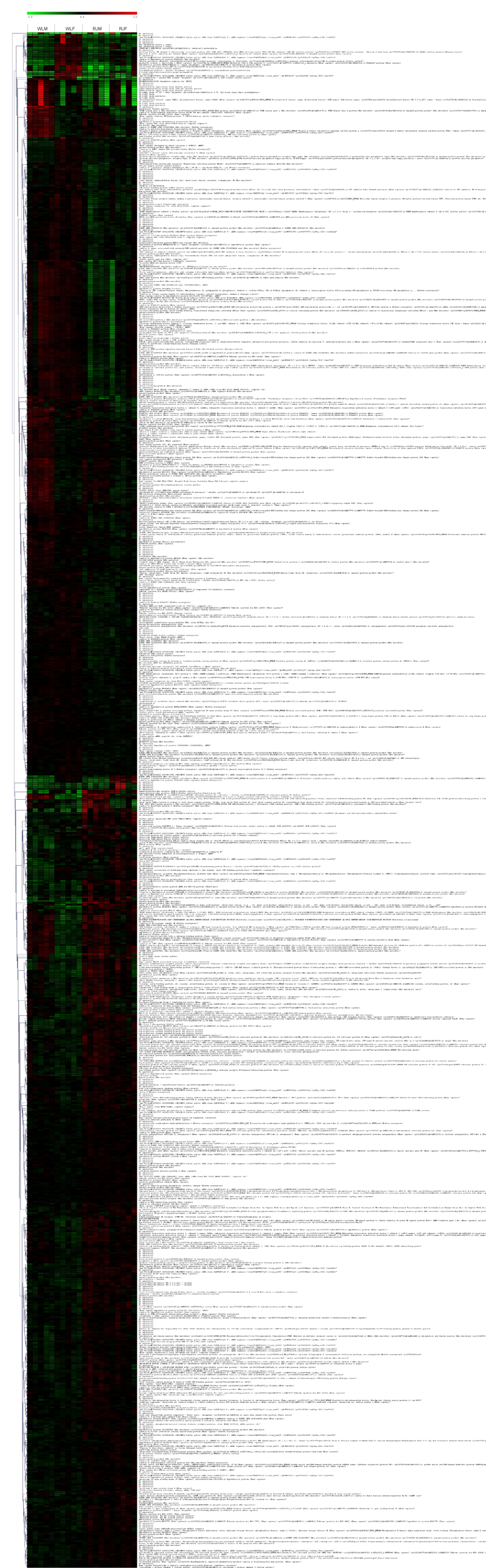

Supplement: Additional File 2 — Hierarchical clustering of microarray data for 837 probes, corresponding to 779 differentially expressed in three contrasts between WL and RJ. [file 1471-2164-8-208-S2.jpeg]

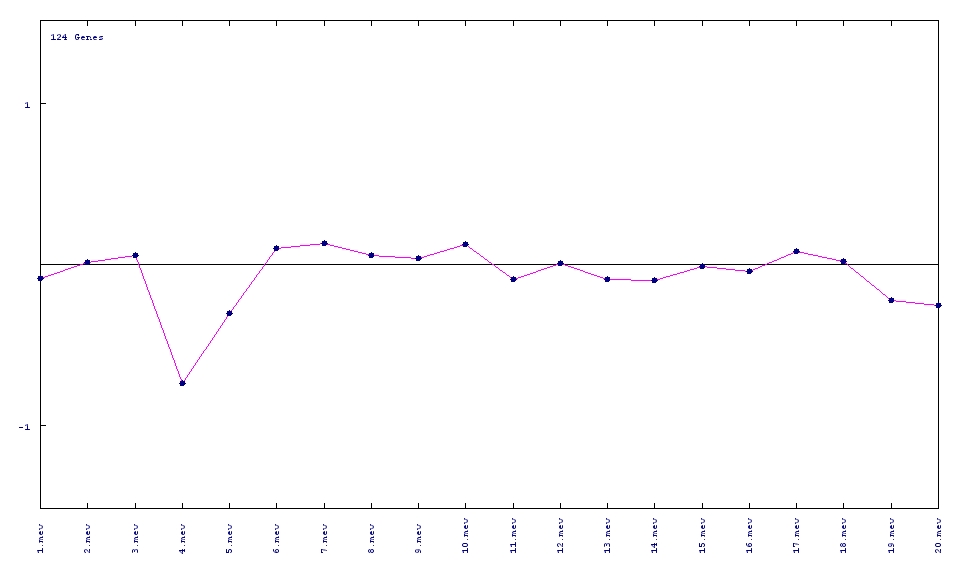

Supplement: Additional File 3 — Along x-axis, individuals included in experiment are presented (1–5 = WL males, 6–10 = WL females, 11–15 = RJ males and 16–20 = RJ females) On the y-axis, mean M-value for sample vs. reference is presented. Mean M-values are based on signal from 124 separate spots on microarray all containing a probe targeting GAPDH (used as an endogenous control in the microarray experiment) [file 1471-2164-8-208-S3.jpeg]

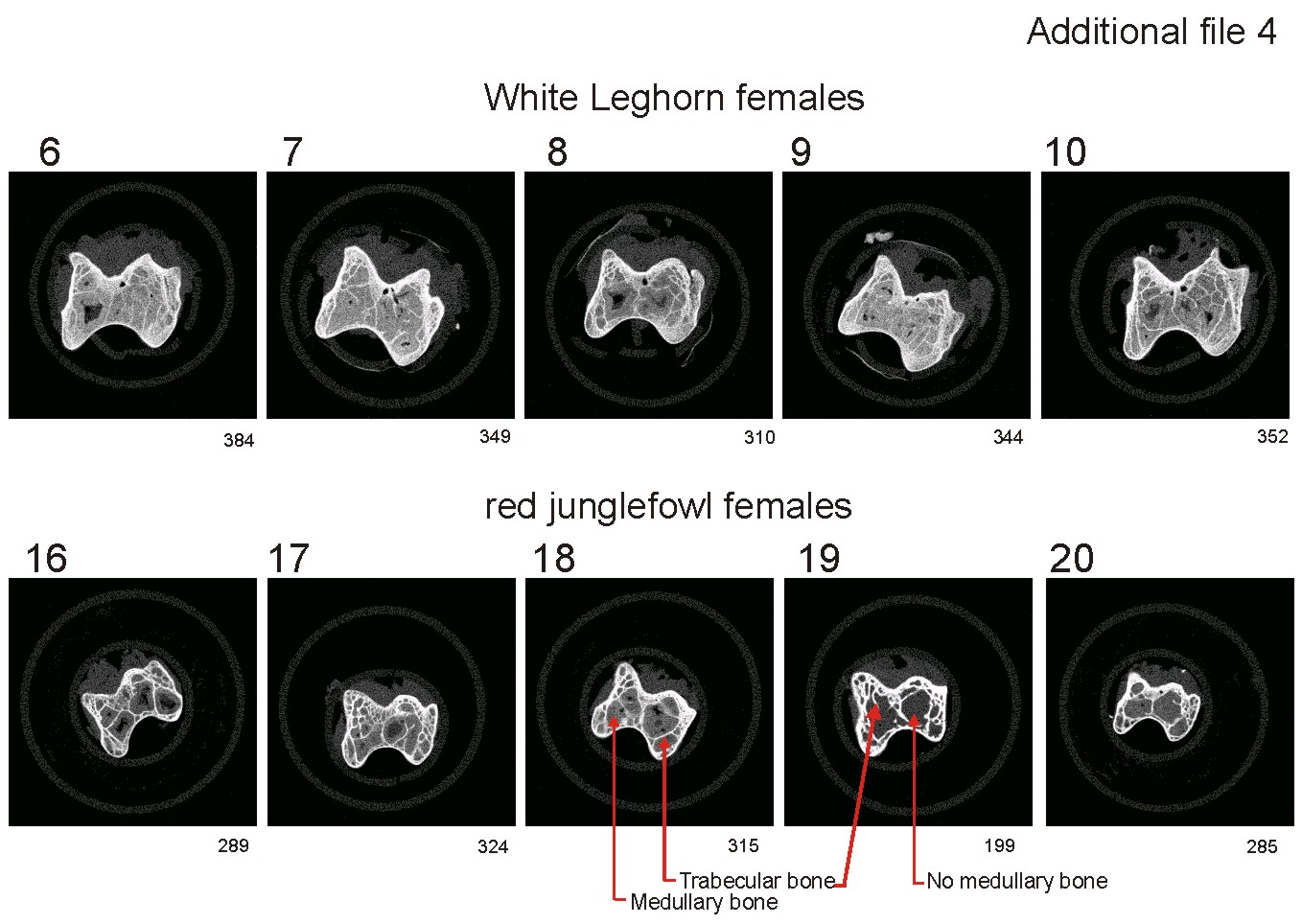

Supplement: Additional File 4 — Metaphyseal images of female femurs from which RNA was derived for the microarray study. Images were derived from phenotyping of the femoral metaphysis by peripheral Quantitative Computerized Tomography (pQCT). The same femoral bones, from which RNA was prepared from the midshaft, were phenotyped in the distal metaphysis by one pQCT-scan at approximately 5% of bone length. Arrows indicate appearance of trabecular bone and medullary bone as well as the absence of medullary bone from red junglefowl female number 19. The noncortical bone mineral density (BMD) in mg/cm3 which in the female metaphysis represents the density of a mix between trabecular and medullary bone is presented below the images corresponding to each individual. The number corresponding to each individual is presented above the images. [file 1471-2164-8-208-S4.jpeg]

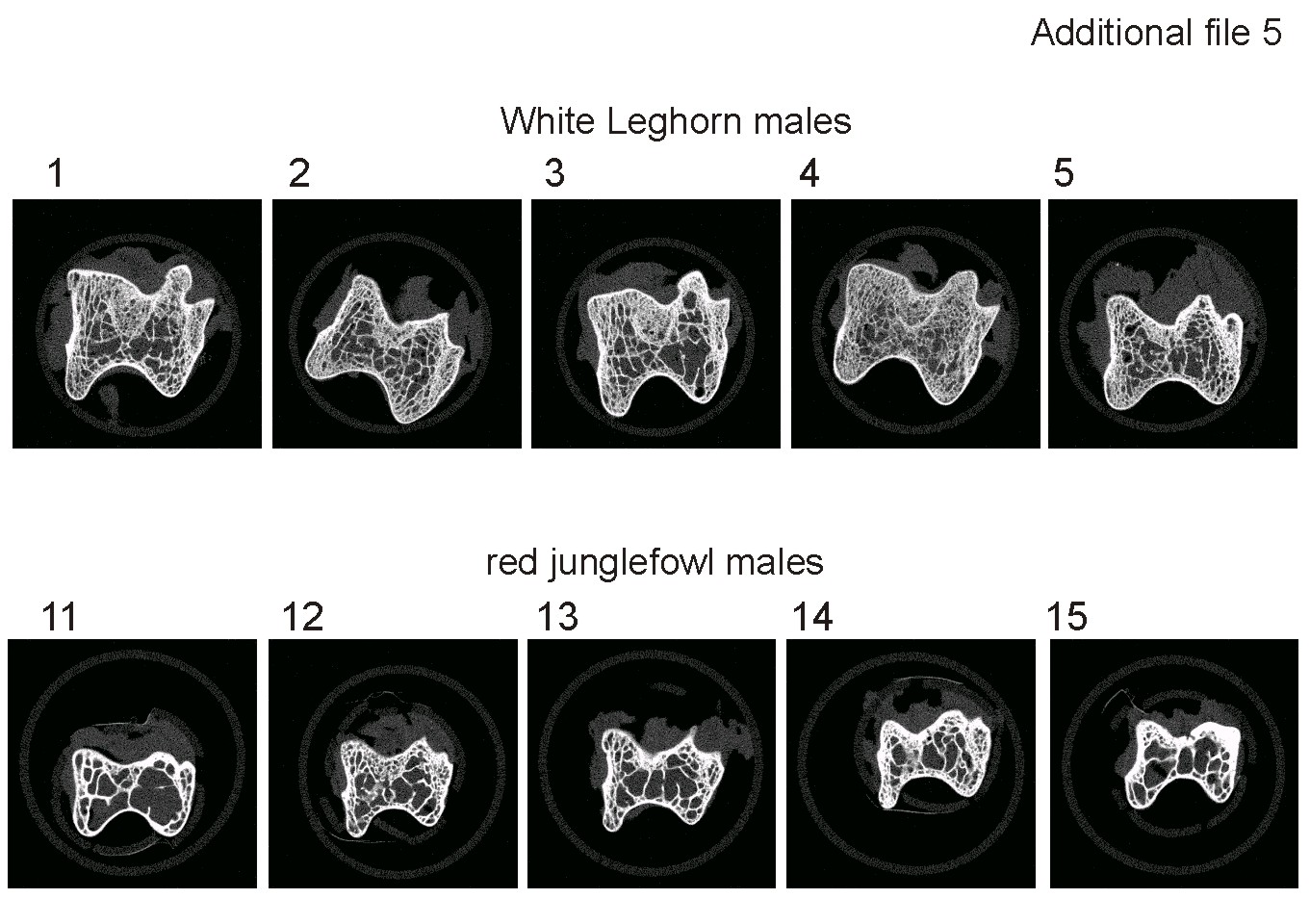

Supplement: Additional File 5 — Metaphyseal images of male femurs from which RNA was derived for the microarray study. Images were derived from phenotyping of the femoral metaphysis by peripheral Quantitative Computerized Tomography (pQCT). The same femoral bones, from which RNA was prepared from the midshaft, were phenotyped in the distal metaphysis by one pQCT-scan at approximately 5% of bone length. The number corresponding to each individual is presented above the images. [file 1471-2164-8-208-S5.jpeg]

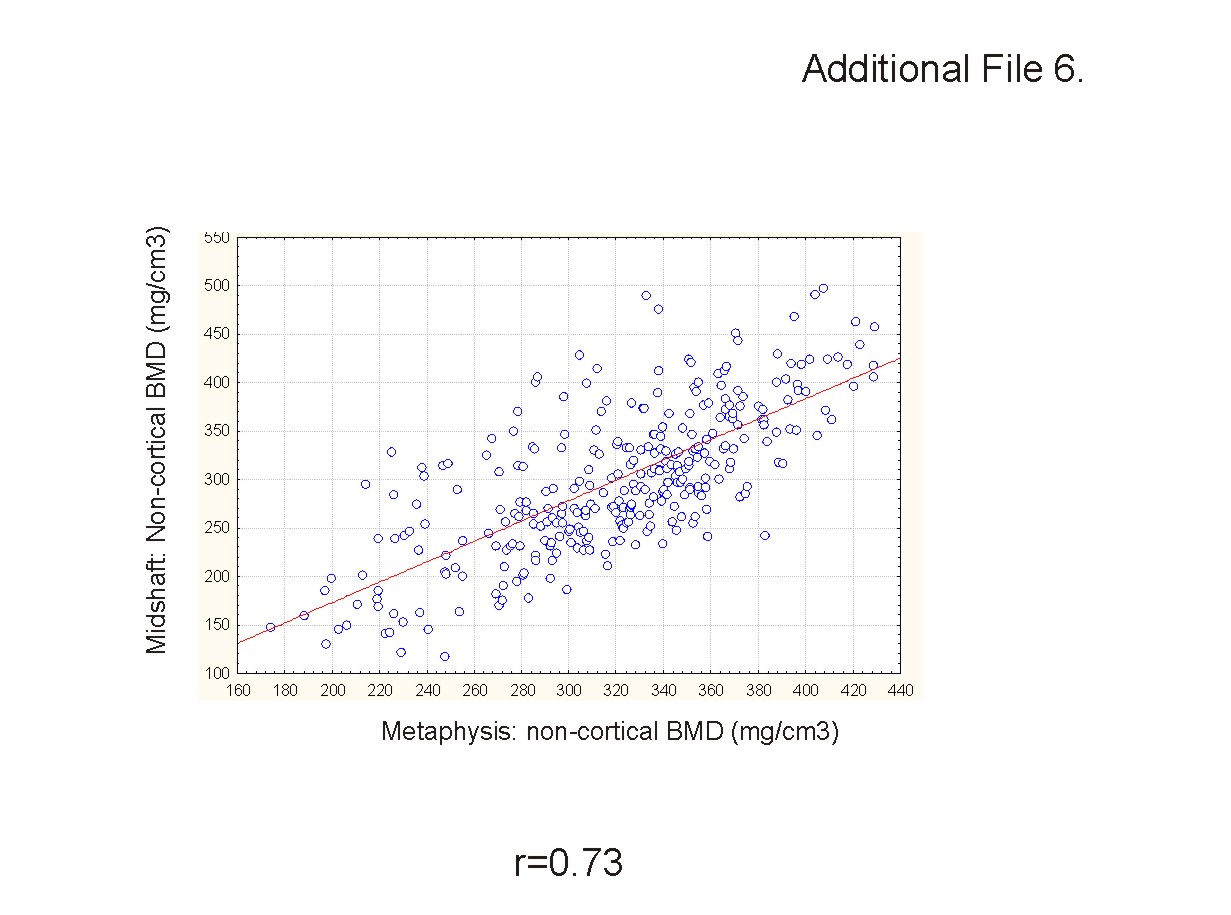

Supplement: Additional File 6 — Correlation between noncortical BMD of the distal femoral metaphysis and medullary BMD of the femoral midshaft. Results are based on an independent sample consisting of 313 female chicken studied at 200 days of age. Phenotyping was performed by peripheral Quantitative Computerized Tomography and noncortical bone was defined by setting the inner thresholds to 600 mg/cm3 and 1000 mg/cm3 at the distal metaphysis and midshaft, respectively. [file 1471-2164-8-208-S6.jpeg]
